# Conformational Dynamics of the Active Site Loop in Dihydroorotase Highlighting the Limitations of Loop-In Structures for Inhibitor Docking

**DOI:** 10.3390/ijms26199688

**Published:** 2025-10-04

**Authors:** Yen-Hua Huang, Tsai-Ying Huang, Man-Cheng Wang, Cheng-Yang Huang

**Affiliations:** 1 Department of Biomedical Sciences, Chung Shan Medical University, Taichung City 40201, Taiwan; 2 The Affiliated Senior High School of National Chung Hsing University, Taichung City 412011, Taiwan; 3 Department of Medical Research, Chung Shan Medical University Hospital, Taichung City 40201, Taiwan

**Keywords:** dihydroorotase, 5-fluoroorotate, myricetin, active site loop, docking, conformational dynamics

## Abstract

Dihydroorotase (DHOase) catalyzes the reversible cyclization of *N*-carbamoyl-L-aspartate to dihydroorotate, a key step in de novo pyrimidine biosynthesis. A flexible active site loop in DHOase undergoes conformational switching between loop-in and loop-out states, influencing substrate binding, catalysis, and inhibitor recognition. In this study, we identified 5-fluoroorotate (5-FOA) and myricetin as inhibitors of *Saccharomyces cerevisiae* DHOase and systematically analyzed 97 crystal structures and AlphaFold 3.0 models of DHOases from 16 species representing types I, II, and III. Our results demonstrate that loop conformation is not universally ligand-dependent and varies markedly across DHOase types, with type II enzymes showing the greatest flexibility. Notably, *S. cerevisiae* DHOase consistently adopted the loop-in state, even with non-substrate ligands, restricting accessibility for docking-based inhibitor screening. Docking experiments with 5-FOA and myricetin confirmed that the loop-in conformation prevented productive active-site docking. These findings highlight the importance of selecting appropriate loop conformations for structure-based drug design and underscore the need to account for loop dynamics in inhibitor screening.

## 1. Introduction

**Figure 1:**
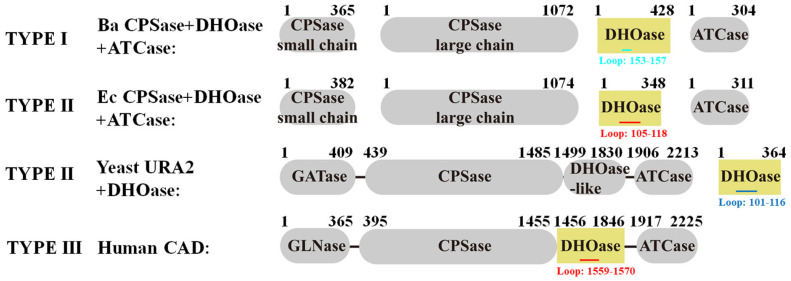
Domain organization of pyrimidine biosynthesis enzymes and the flexible loop region in DHOase across different species. Type I enzymes (e.g., DHOase from *Bacillus anthracis*, Ba) have separate CPSase, DHOase, and ATCase subunits. Type II enzymes (e.g., DHOase from *E. coli*, Ec) also have separate CPSase, DHOase, and ATCase subunits. However, type II DHOases are smaller. In yeast type II (e.g., DHOase from *S. cerevisiae*), DHOase is encoded separately from the multifunctional URA2 protein that harbors CPSase and ATCase functional domains. Type III (e.g., human CAD) integrates CPSase, DHOase, and ATCase domains into a single polypeptide. The DHOase domains (highlighted in yellow) include the flexible active site loops (marked in cyan, red, or blue) with their respective residue ranges indicated. Loop regions are color-coded: red for loop-out state (*E. coli* DHOase: residues 105–118; human DHOase: residues 1559–1570), cyan for the shorter loop which may restrict movement (*B. anthracis* DHOase: residues 153–157), and blue for loop-in state (*S. cerevisiae* DHOase: residues 101–116).

The de novo pyrimidine biosynthesis pathway is essential for all living organisms, as it provides the fundamental building blocks, uridine monophosphate (UMP) and its derivatives, which are required for the synthesis of RNA, DNA, phospholipids, glycoproteins, and glycogen [1,2,3,4,5]. A key enzyme in this pathway is dihydroorotase (DHOase; EC 3.5.2.3), a zinc-dependent metalloenzyme that catalyzes the reversible intramolecular cyclization of *N*-carbamoyl-L-aspartate (NCA) to dihydroorotate (DHO), representing the third step in the sequential conversion of precursors into pyrimidine nucleotides [6,7,8,9,10,11,12,13,14,15,16,17,18]. This reaction bridges the upstream condensation of carbamoyl phosphate and aspartate with the downstream oxidation of DHO by dihydroorotate dehydrogenase [19]. Given its central role, inhibition of this pathway has demonstrated therapeutic potential in cancer treatment by reducing tumor cell proliferation and inducing apoptosis [1,20,21,22,23], as well as in antiparasitic therapies targeting pathogens such as *Toxoplasma gondii* [24] and *Plasmodium falciparum* [25,26,27,28,29]. While the catalytic function of DHOase is evolutionarily conserved, its structural organization varies significantly across species (Figure 1). In prokaryotes such as *Escherichia coli*, DHOase typically exists as a monofunctional protein [30,31,32,33]. In contrast, eukaryotic DHOase, including that from *Homo sapiens*, is integrated into the multifunctional CAD enzyme, which combines the activities of carbamoyl phosphate synthetase (CPSase), aspartate transcarbamoylase (ATCase), and DHOase within a single polypeptide chain [34,35,36,37,38]. In yeast, such as *Saccharomyces cerevisiae*, DHOase is encoded separately from the bifunctional CPSase-ATCase gene (URA2) [39,40,41,42,43,44,45,46], further illustrating the architectural diversity across species. Despite their conserved function, DHOases from different organisms often exhibit low sequence identity, reflecting significant evolutionary divergence [14,47]. Accordingly, DHOases have been classified into three distinct types [14]. Type I DHOases, considered evolutionarily ancient and larger in size (approximately 45 kDa), are typically found in Gram-positive bacteria. Type II DHOases are smaller (approximately 38 kDa) and are present in most eubacteria (e.g., *E. coli*), fungi (e.g., *S. cerevisiae*), and plants. Type III DHOase refers to the DHOase domain found within the multifunctional CAD protein in mammals (e.g., human DHOase). The evolutionary rationale behind these structural and organizational differences among DHOase types remains an intriguing subject for further investigation.

A hallmark structural feature of DHOase is the flexible loop near the active site, which undergoes substantial conformational changes during the catalytic cycle [6]. Traditionally, this loop has been categorized into two major conformational states: the loop-in conformation, which is associated with substrate binding and transition state stabilization, and the loop-out conformation, which is associated with product release and non-substrate ligand or inhibitor binding [16,48,49]. This binary model is primarily supported by structural studies of type II bacterial DHOases, especially the well-characterized *E. coli* enzyme. However, recent high-resolution structural analyses challenge this model. Several non-substrate ligands and inhibitors, including malate [9,39,50], 5-fluoroorotate (5-FOA) [40], 5-aminouracil [42], 5-fluorouracil [9,42], and plumbagin [41], have been observed binding to DHOase active sites while the loop adopts a loop-in conformation. Notably, in *S. cerevisiae* DHOase, 5-FOA, which was previously assumed to mimic product release in the loop-out conformation, binds in a loop-in state [40]. This emerging evidence suggests that loop dynamics are more complex and variable than previously recognized and underscores the need for systematic analysis across available structures.

Advances in protein dynamics have reshaped classical models of enzyme function, challenging static representations of catalytic intermediates [51,52,53,54,55,56,57,58,59,60,61,62,63]. The concept of conformational ensembles proposes that enzymes populate multiple pre-existing conformations, with ligand binding shifting the equilibrium rather than inducing a singular conformational change. This model is particularly relevant for DHOase, where the flexible active site loop plays a critical role in substrate recognition and catalysis. Whether the loop-in conformation is universally required for substrate binding, or whether alternative states exist across different DHOase types and species, remains an open question that warrants comprehensive structural investigation.

In this study, we identified 5-FOA and myricetin as inhibitors of *S. cerevisiae* DHOase. Unexpectedly, although the loop-in conformation is generally associated with enhanced ligand interactions [6], docking simulations did not predict high-affinity binding of either 5-FOA or myricetin within the active site of *S. cerevisiae* DHOase. This limitation likely arises from steric hindrance imposed by the loop-in state, which may restrict ligand access. Notably, all ten available *S. cerevisiae* DHOase crystal structures exhibit loop-in conformations, with no evidence of a loop-out state. This steric restriction extends to other DHOase structures adopting loop-in conformations, rendering them unsuitable as templates for docking-based inhibitor screening. To address this, we systematically analyzed the conformational states of the active site loop across 97 DHOase crystal structures from 16 species, spanning types I, II, and III. Structures were categorized based on loop conformation (loop-in vs. loop-out) and ligand binding status. Our findings reveal significant variability in loop dynamics across DHOase types and species. Moreover, AlphaFold 3.0 predictions further highlighted discrepancies between modeled and experimental loop states in some DHOases, indicating the complexity of flexible loop regions. Overall, this study underscores the importance of considering loop dynamics in structure-based inhibitor design, particularly when targeting the loop-in state, as exemplified by myricetin’s inhibition of *S. cerevisiae* DHOase.

## 2. Results

### 2.1. Identification of 5-FOA and Myricetin as Inhibitiors of Yeast DHOase

**Figure 2:**
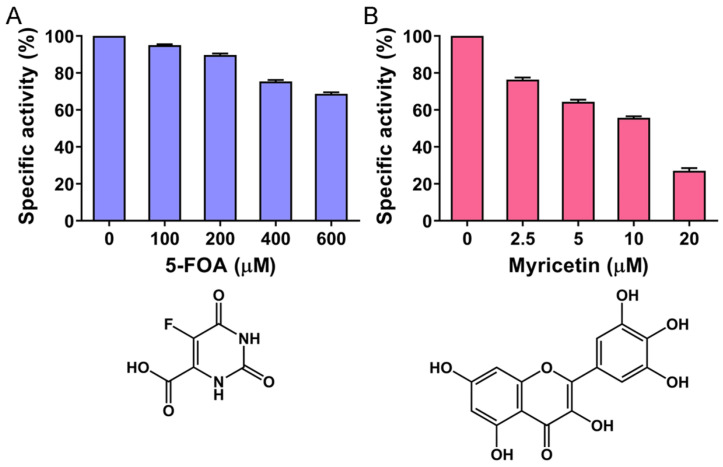
Identification of 5-FOA and myricetin as inhibitors of yeast DHOase from *S. cerevisiae*. Under standard assay conditions, varying concentrations of (**A**) 5-FOA and (**B**) myricetin were tested for their inhibitory effects on *S. cerevisiae* DHOase activity. Graphical analysis revealed an IC_50_ of 12.48 ± 0.47 μM for myricetin, whereas 5-FOA exhibited an IC_50_ value exceeding 600 μM. Error bars represent the standard deviation from three independent measurements.

5-Fluoroorotic acid (5-FOA) is a known active-site inhibitor of type II DHOase, such as those from *E. coli* [49] and *P. falciparum* [29], as well as type III DHOase from humans [41]. Myricetin, a flavonol, has also been reported as a competitive inhibitor of *Klebsiella pneumoniae* DHOase (a type II enzyme) [12]. Despite these known inhibitory activities, both 5-FOA and its analog 5-aminoorotate have shown no inhibition against *Bacillus anthracis* DHOase [64,65], likely due to significant structural and biochemical differences across DHOase types (Figure 1). It remained unclear whether these DHOase inhibitors could effectively inhibit *S. cerevisiae* DHOase, a eukaryotic enzyme previously classified as type II based on biochemical properties. Although *S. cerevisiae* is a eukaryote, its DHOase shares more catalytic characteristics with bacterial type II enzymes [46,47]. In prior work, we determined the crystal structure of the *S. cerevisiae* DHOase complexed with 5-FOA, indicating binding capability [40]. However, the inhibitory effect of 5-FOA on enzymatic activity had not been experimentally validated until this study. For reference, 200 μM 5-FOA is known to reduce human DHOase activity by 26% [41], and 40 μM myricetin inhibits *K. pneumoniae* DHOase by 50% [12]. To test these effects in *S. cerevisiae* DHOase, we performed standard enzymatic assays using varying concentrations of both inhibitors (Figure 2). Our results showed that 5-FOA at 100, 200, 400, and 600 μM inhibited *S. cerevisiae* DHOase by 5%, 10%, 23%, and 33%, respectively (Figure 2A). Similarly, myricetin at 2.5, 5, 10, and 20 μM resulted in 22%, 35%, 43%, and 71% inhibition, respectively (Figure 2B). The half-maximal inhibitory concentration (IC_50_) was determined through graphical analysis to be 12.48 ± 0.47 μM for myricetin, while 5-FOA failed to reach 50% inhibition even at 600 μM. These findings demonstrate that both 5-FOA and myricetin also inhibit *S. cerevisiae* DHOase.

### 2.2. Dynamic Binding Modes of 5-FOA to DHOase

**Figure 3:**
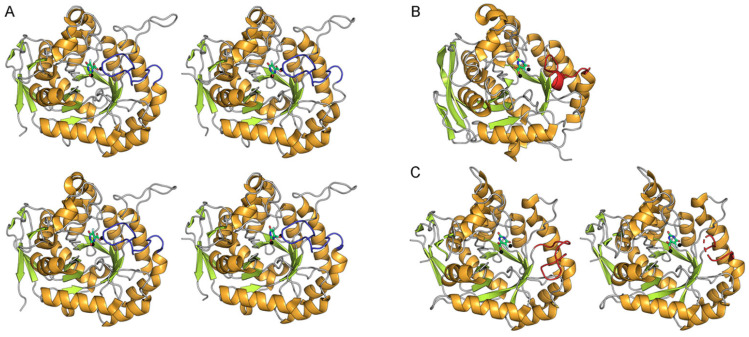
Distinct 5-FOA binding modes across different DHOase species: (**A**) *S. cerevisiae*, (**B**) human, and (**C**) *E. coli*. Different monomers from the solved DHOase structures are shown, including tetrameric *S. cerevisiae* (PDB ID 7CA0), monomeric human DHOase (PDB ID 4C6L), and dimeric *E. coli* DHOase (PDB ID 2EG8). The flexible active site loop is shown in red when adopting the loop-out conformation, and in blue when engaging the ligand in the loop-in conformation. The bound inhibitor 5-FOA is shown in lime green, and the metal ions are depicted as black spheres. In *E. coli* DHOase, the dashed line represents an unresolved region in the structure, possibly reflecting the dynamic transition of the loop. The loop residues are as follows: *S. cerevisiae* DHOase (residues 102–116), human DHOase (residues 1560–1569), and *E. coli* DHOase (residues 106–118).

A defining structural feature of DHOase is its flexible active site loop, which undergoes conformational changes throughout the catalytic cycle [6,16,49]. In the tetrameric *S. cerevisiae* DHOase (PDB ID: 7CA0), all four monomers bind 5-FOA via the loop-in conformation (Figure 3A). In contrast, the monomeric human DHOase (PDB ID: 4C6L) exhibits a loop-out conformation upon 5-FOA binding (Figure 3B). Interestingly, the dimeric *E. coli* DHOase (PDB ID: 2EG8) shows an asymmetric binding pattern, with one subunit in a clear loop-out conformation and the other in an undefined state that may represent a partially folded loop-out conformation (Figure 3C). The observed differences in binding modes involving the dynamic active site loop raise an intriguing question as to whether the loop contributes to ligand specificity and recognition. To date, no complex structure of 5-FOA bound to type I DHOases (e.g., from *B. anthracis* or *Staphylococcus aureus*) has been reported. Given the lack of inhibitory activity of 5-FOA against *B. anthracis* DHOase [64,65], it would be valuable to investigate its binding either experimentally or through computational modeling in order to identify structural determinants of inhibition resistance and to elucidate broader binding principles across DHOase types.

### 2.3. Docking Analysis of Myricetin and 5-FOA to S. cerevisiae DHOase

**Figure 4:**
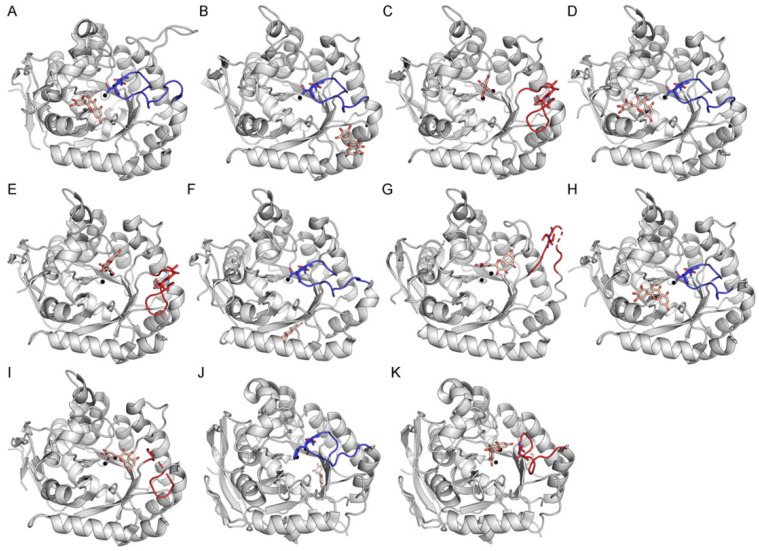
Docking analysis of myricetin to DHOase. Representative DHOase structures with defined loop conformations were used for docking simulations with myricetin. (**A**) *S. cerevisiae* DHOase (PDB ID: 6L0A) in the loop-in conformation. (**B**) *E. coli* DHOase (PDB ID: 2EG7), monomer in the loop-in conformation. (**C**) *E. coli* DHOase (PDB ID: 2EG7), monomer in the loop-out conformation. (**D**) *Salmonella enterica* DHOase (PDB ID: 3JZE), monomer in the loop-in conformation. (**E**) *S. enterica* DHOase (PDB ID: 3JZE), monomer in the loop-out conformation. (**F**) *Campylobacter jejuni* DHOase (PDB ID: 3PNU), monomer in the loop-in conformation. (**G**) *C. jejuni* DHOase (PDB ID: 3PNU), monomer in the loop-out conformation. (**H**) *Yersinia pestis* DHOase (PDB ID: 6CTY), monomer in the loop-in conformation. (**I**) *Y. pestis* DHOase (PDB ID: 6CTY), monomer in the loop-out conformation. (**J**) Human DHOase (PDB ID: 8GVZ), loop-in conformation. (**K**) Human DHOase (PDB ID: 4C6C), loop-out conformation. The flexible active site loop is shown in red for loop-out conformations and in blue for loop-in conformations. Myricetin is represented in melon, and metal ions are depicted as black spheres. The two key residues involved in substrate binding and product release, namely Thr109 and Thr110 in *E. coli* DHOase or their corresponding residues in other DHOases, are also highlighted using stick representation in the structural models. Dashed lines indicate unresolved loop regions, possibly reflecting loop flexibility or transitions between conformational states.

**Figure 5:**
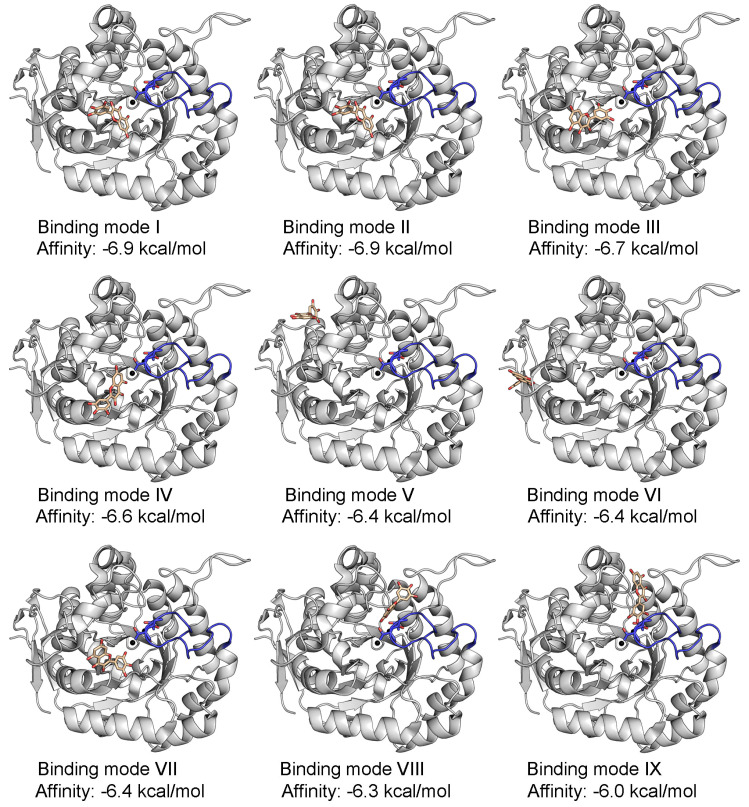
Docking results of myricetin to *S. cerevisiae* DHOase. Nine different binding poses were predicted by AutoDock Vina. All poses failed to meet the criteria for successful docking, as none involved interactions with the substrate-binding or metal-binding residues.

**Figure 6:**
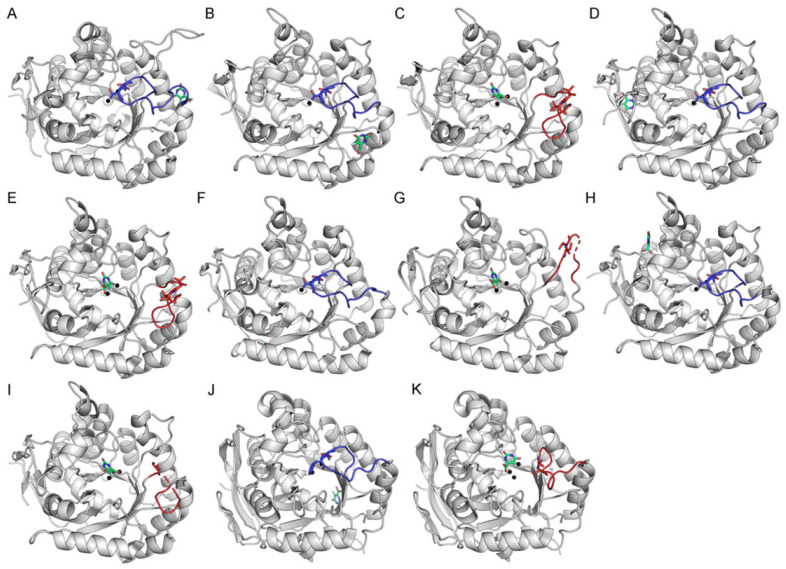
Docking analysis of 5-FOA to DHOase. Representative DHOase structures with defined loop conformations were used for docking simulations with 5-FOA. (**A**) *S. cerevisiae* DHOase (PDB ID: 6L0A) in the loop-in conformation. (**B**) *E. coli* DHOase (PDB ID: 2EG7), monomer in the loop-in conformation. (**C**) *E. coli* DHOase (PDB ID: 2EG7), monomer in the loop-out conformation. (**D**) *S. enterica* DHOase (PDB ID: 3JZE), monomer in the loop-in conformation. (**E**) *S. enterica* DHOase (PDB ID: 3JZE), monomer in the loop-out conformation. (**F**) *C. jejuni* DHOase (PDB ID: 3PNU), monomer in the loop-in conformation. (**G**) *C. jejuni* DHOase (PDB ID: 3PNU), monomer in the loop-out conformation. (**H**) *Y. pestis* DHOase (PDB ID: 6CTY), monomer in the loop-in conformation. (**I**) *Y. pestis* DHOase (PDB ID: 6CTY), monomer in the loop-out conformation. (**J**) Human DHOase (PDB ID: 8GVZ), loop-in conformation. (**K**) Human DHOase (PDB ID: 4C6C), loop-out conformation. The flexible active site loop is shown in red for loop-out conformations and in blue for loop-in conformations. 5-FOA is represented in lime green, and metal ions are depicted as black spheres. The two key residues involved in substrate binding and product release (Thr109 and Thr110 in *E. coli* DHOase, or their corresponding residues in other DHOases) are also highlighted using stick representation in the structural models. Dashed lines indicate unresolved loop regions, possibly reflecting loop flexibility or transitions between conformational states.

Myricetin was identified as an inhibitor of *S. cerevisiae* DHOase in this study (Figure 2B). Therefore, it is of interest to determine how myricetin binds to the active site to exert its inhibitory effect. AutoDock Vina, a widely utilized docking tool for predicting compound–protein interactions, was employed to explore potential binding poses of myricetin at the active site of *S. cerevisiae* DHOase (Figure 4). Success is defined as docking that occurs within the active site, while failure is defined as docking that does not involve any active site residues, including the substrate-binding and metal-binding residues. Surprisingly, myricetin could not be docked into the active site of *S. cerevisiae* DHOase (Figure 4A). Nine different binding poses were predicted by AutoDock Vina, but none met the criteria for successful docking, as they did not involve interactions with the substrate-binding or metal-binding residues (Figure 5). Similarly, although the crystal structure of *S. cerevisiae* DHOase complexed with 5-FOA has been previously solved (PDB ID: 7CA0; structure No. 76 in Table 1), 5-FOA could not be docked into the active site (Figure 6A). This may be due to the steric hindrance imposed by the active site loop, which adopts a loop-in conformation in all available *S. cerevisiae* DHOase structures. Indeed, all ten currently available crystal structures of this enzyme (Structure Nos. 68–77 in Table 1) exhibit the loop-in conformation, with no evidence of a loop-out state. These observations suggest that the loop-in conformation may block access to the binding site in docking simulations, leading to inaccurate predictions for ligands like myricetin and 5-FOA. This issue highlights the importance of considering loop dynamics in structure-based inhibitor design and underscores the need for further structural and biochemical studies to better model ligand interactions under different conformational states.

### 2.4. Docking Analysis of Myricetin and 5-FOA to DHOases Exhibiting Both Loop-In and Loop-Out Conformations

We found that the loop-in conformation is generally unsuitable for docking analysis, as exemplified by attempts to dock the inhibitors myricetin (Figure 5) and 5-FOA (Figure 6A) into *S. cerevisiae* DHOase. Since DHOase is a critical target for anticancer [1,23,41,66] and antipathogenic [12,50,67,68] drug development, it is essential to carefully select structural templates that are suitable for docking experiments. To determine whether the docking results from *S. cerevisiae* DHOase can be generalized to other DHOases, we performed docking experiments using DHOases with available crystal structures in both loop-in and loop-out conformations. These included DHOases from *E. coli* (Figure 4B,C and Figure 6B,C), *Salmonella enterica* subsp. enterica serovar Typhimurium str. LT2 (Figure 4D,E, and Figure 6D,E), *Campylobacter jejuni* (Figure 4F,G and Figure 6F,G), *Yersinia pestis* (Figure 4H,I and Figure 6H,I), and human DHOase (Figure 4J,K and Figure 6J,K). Similar to *S. cerevisiae* DHOase, the loop-in conformation of these enzymes prevented successful docking of myricetin and 5-FOA into the active site. In contrast, the loop-out conformations of all examined DHOases allowed successful docking, with the ligands interacting with the conserved substrate-binding residue Arg (e.g., Arg20 in *E. coli* DHOase).

Although the loop-in conformation is believed to enhance binding affinity through interactions with loop residues, as demonstrated in the crystal structure and the mutational analysis of *S. cerevisiae* DHOase [40], where Thr105 and Thr106 stabilize the inhibitor 5-FOA, this conformation introduces steric hindrance that can obstruct ligand access during docking simulations (Figure 6A). Therefore, structural templates in docking analyses of DHOase–ligand interactions should initially favor the loop-out conformation. To improve predictive accuracy, the loop can subsequently be manually repositioned into the loop-in conformation to account for its potential contributions to ligand binding during structural modeling.

### 2.5. Comparative Analysis of Active Site Loop Conformations Across DHOase Types Using Crystal Structures from the Protein Data Bank

**Figure 7:**
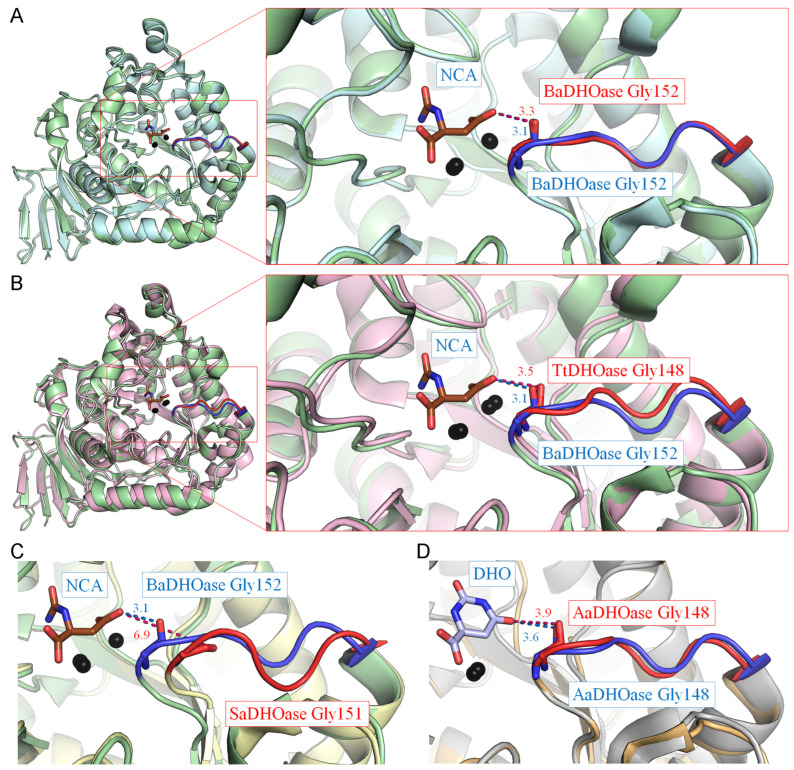
Classification of loop-in conformations in type I DHOases without bound ligands. (**A**) Structural superimposition of *B. anthracis* DHOase (BaDHOase) with (PDB ID: 4YIW) and without (PDB ID: 3MPG) the ligand NCA. The loop in the ligand-bound structure is colored blue, while the corresponding loop in the ligand-free structure is in red. Although the interacting residue Gly does not form side-chain contacts with the ligand, the peptide backbone oxygen (also in red) maintains a comparable position in both structures. Due to the nearly identical loop conformations, the unbound structure was also classified as having a loop-in conformation. (**B**) Structural comparison of BaDHOase-NCA complex with *T. thermophilus* DHOase (TtDHOase; PDB ID: 2Z00). Based on the similar loop conformation, the ligand-free TtDHOase structure was classified as loop-in. (**C**) Structural superimposition of BaDHOase-NCA complex with *S. aureus* DHOase (SaDHOase; PDB ID: 3GRI). Although both enzymes share similar loop lengths, the putative ligand-interacting residue Gly151 in SaDHOase is located 6.9 Å away from the ligand-binding site, indicating that the loop cannot interact with the ligand in this conformation. Thus, this loop was classified as loop-out. (**D**) Structural superimposition of *A. aeolicus* DHOase (AaDHOase) with (PDB ID: 4BJH) and without (PDB ID: 1XRF) NCA. Due to the highly similar loop conformations and conserved positioning of the ligand-interacting residue Gly148, the ligand-free structure of AaDHOase was also categorized as loop-in.

Despite the fact that ligand interaction, such as 5-FOA binding to the loop, can chemically enhance affinity as demonstrated in *S. cerevisiae* DHOase [40], the loop does not necessarily interact with the inhibitor in all species. In contrast to *S. cerevisiae*, the loop in human and *E. coli* DHOases does not interact with 5-FOA (Figure 3). This observation raises the question of whether loop conformation is universally associated with ligand binding and warrants a comprehensive structural comparison of DHOases across different organisms. To date, no systematic structural analysis has classified loop conformations across all available DHOase structures. Many recent findings challenge the classical model derived from *E. coli*, where the loop-in conformation is linked to substrate binding (NCA) and the loop-out conformation to product release (DHO) or non-substrate ligand binding (e.g., 5-FOA). This underscores the need to reassess the model. Specifically, determining the loop conformation (in vs. out) in various DHOase types and states is crucial for understanding active site accessibility and for structure-based drug design targeting DHOase (Table 1). Accordingly, we analyzed a total of 97 DHOase structures from 16 species deposited in the Protein Data Bank. These include Type I enzymes from *Porphyromonas gingivalis*, *Methanococcus jannaschii* [69], *Thermus thermophilus*, *Aquifex aeolicus* [70,71,72,73,74], *S. aureus*, and *B. anthracis* [64]; Type II from *S. cerevisiae* [39,40,42], *Campylobacter jejuni*, *E. coli* [16,49,75], *Salmonella enterica* subsp. enterica serovar Typhimurium LT2, *Yersinia pestis* [50], *Burkholderia cenocepacia*, and *Vibrio cholerae* [50]; and Type III from *Homo sapiens* [10,14,76,77]. Structures from *Agrobacterium fabrum* (no. 10) and *Chaetomium thermophilum* (no. 44) were not included in the analysis (Table 1). *A. fabrum* DHOase (PDB ID: 2OGJ; sequence ID: WP_010972896.1) was excluded from the loop classification because it could not be structurally superimposed with DHOases, such as *B. anthracis* DHOase (Supplementary Materials Figure S1). Furthermore, it lacks the highly conserved Arg residue essential for substrate binding (e.g., Arg20 in *E. coli*, Arg63 in *B. anthracis*, Arg65 in *A. aeolicus*), which is replaced by Trp81 in *A. fabrum* (Supplementary Materials Figure S2), suggesting that this protein may not be a true DHOase. In fact, an incorrectly annotated DHOase from *Agrobacterium tumefaciens* C58 has also been identified previously [78]. Similarly, the inactive DHOase-like domain from *C. thermophilum* CAD (PDB ID: 5NNL) was excluded due to lack of enzymatic activity. Each structure was assessed for ligand binding status, length (number of residues), sequence identity (%), structural similarity (TM-score) [79], root-mean-square deviation (RMSD) [79], DHOase type, and loop conformation. Loop-in was assigned when the loop directly interacted with a bound ligand; loop-out was assigned when no such interaction was present, regardless of ligand binding. Some type I DHOase structures without bound ligands are still classified as exhibiting the loop-in conformation, due to their high structural similarity to known loop-in states (Figure 7 and Table 2), despite the absence of ligands in the active site (see below). All monomers were individually evaluated, whether ligand-bound or apo. Interestingly, although both *S. cerevisiae* and *E. coli* DHOases are classified as type II enzymes, they exhibit markedly different loop behaviors. All 40 monomers across 10 *S. cerevisiae* structures display a loop-in conformation (Table 2), whereas 28 *E. coli* monomers—21 of which are ligand-bound—exhibit a loop-out conformation (Table 3). In type III human DHOase, 52 monomers adopt the loop-out state, including 17 bound to ligands (Table 3). Overall, these findings suggest that loop conformation is not strictly ligand-dependent. The structural diversity among DHOase types highlights the need for a more nuanced understanding of loop dynamics and supports a reevaluation of previously held assumptions about their catalytic mechanisms and druggability.

For type I DHOases, which include representatives such as *A. aeolicus*, *B. anthracis*, *S. aureus*, *P. gingivalis*, and *M. jannaschii*, the majority of structures, including those with PDB IDs 1XRF, 1XRT, 3MPG, 4YIW, and 6GDF, exhibited loop-in conformations, even in the absence of bound ligands (Figure 7; see below). In addition, many type I DHOases possess loops that are too short (6 amino acid residues) to clearly determine whether they adopt a loop-in or loop-out conformation. This structural feature may suggest a predisposition toward the loop-in state in this class. Nonetheless, a few structures, such as 3GRI and 7UOF, showed loop-out conformations. Several entries within this group exhibited disordered or unresolved loop regions, likely reflecting intrinsic structural flexibility in the crystal form, which precluded definitive loop classification.

Type II DHOases, including those from *E. coli*, *S. enterica*, *C. jejuni*, *Y. pestis*, and *S. cerevisiae*, displayed the highest degree of conformational heterogeneity among the three types. In *E. coli* DHOase structures (e.g., PDB IDs 1XGE, 2EG7, 2Z25), asymmetric loop states were observed within dimers, where one monomer adopted a loop-in conformation and the other loop-out. This suggests dynamic loop switching during the catalytic cycle. In contrast, *S. cerevisiae* DHOase consistently exhibited loop-in conformations across all four monomers of its tetrameric unit (e.g., in structures 6L0A, 6L0B, 7CA0, 7CA1), regardless of the type of bound ligand. Other bacterial type II DHOases, such as those from *Salmonella* and *Campylobacter*, also displayed both loop-in and loop-out conformations, depending on ligand presence. These findings indicate that type II DHOases exhibit the broadest spectrum of loop conformational states, supporting their role in adaptive binding and conformational flexibility. The strong preference for the loop-in conformation observed in yeast DHOase, even in the presence of non-substrate ligands, has important implications for inhibitor design and the prediction of drug binding modes. In addition, unlike other type II DHOases, yeast DHOase interacts with a CAD-like protein [45,46], whereas bacterial type II DHOases, such as *E. coli* DHOase, function independently in conjunction with partner proteins CPSase and ATCase [2]. Whether this reflects a distinct evolutionary adaptation remains to be investigated.

Type III DHOases, exemplified by the human CAD DHOase domain, displayed loop conformations that appear to be ligand-dependent. Loop-out conformations were observed predominantly in apo structures (e.g., 4C6C, 6HFE) and in complexes with product-like inhibitors such as 5-FOA (e.g., 4C6L, 8PBN). In contrast, loop-in conformations were stabilized in structures bound to substrate or ligand, as seen in PDB IDs 6HFR, 8GVZ, and 8PBH. These conformational differences suggest that type III DHOases undergo ligand-driven switching between loop-in and loop-out states, with the loop-in conformation likely associated with catalytically competent states. This behavior underscores the therapeutic potential of targeting loop dynamics for selective drug development.

Across all 95 analyzed DHOase structures, loop-in conformations were identified in approximately 45% of cases, notably in structures from *S. cerevisiae*, *Y. pestis*, and human DHOase bound to substrates. Loop-out conformations were observed in about 40% of structures, particularly in *E. coli* and apo-human DHOase. The remaining ~15% of structures had loops that were disordered or structurally unresolved, making conformation assignment difficult. Notably, asymmetry in loop conformation was frequently observed in dimeric DHOases, such as those from *E. coli* and *Salmonella*, where different monomers within the same dimer adopted distinct loop states. This suggests possible cooperativity of DHOase with the partner protein(s) or conformational selection mechanisms that may regulate enzyme activity during the catalytic cycle.

### 2.6. Classification of the Loop Conformation States in Type I DHOases

Despite evolutionary divergence, the flexible active site loop which acts as a lid to regulate catalysis and substrate binding is a conserved feature across DHOases from *E. coli* to humans. However, in many type I DHOases, the loop length is notably shorter than in types II and III, often complicating efforts to classify its conformation as either loop-in or loop-out (Table 2; DHOases are listed according to loop length in amino acid residues). Interestingly, the loop length appears to correlate with enzyme type: type II DHOases typically have loops of 14–16 residues, type III (human) DHOase features a 12-residue loop, and type I DHOases generally exhibit shorter loops ranging from 6 to 12 residues. Notably, two type I enzymes, *M. jannaschii* (12 residues) and *P. gingivalis* (11 residues), possess relatively longer loops. Whether these enzymes exhibit catalytic properties more akin to type I or II DHOases remains to be further investigated.

To explore the conformational diversity of active site loops among type I DHOases, we examined representative structures from *B. anthracis* (BaDHOase), *T. thermophilus* (TtDHOase), *S. aureus* (SaDHOase), and *A. aeolicus* (AaDHOase) (Figure 7). Although several of these structures lack bound ligands, structural superimpositions with ligand-bound models were performed to assess whether their loop conformations resembled loop-in or loop-out states. For BaDHOase, comparison between ligand-bound (PDB ID: 4YIW) and ligand-free (PDB ID: 3MPG) structures revealed nearly identical loop conformations. Although no direct side-chain interaction with the ligand was observed, the backbone oxygen atom of a loop glycine residue occupied a similar spatial position in both structures (Figure 7A). Consequently, the ligand-free BaDHOase was classified as adopting a loop-in conformation. This observation may suggest that BaDHOase intrinsically favors the loop-in state, although further structural and biochemical investigations are required to confirm whether a loop-out conformation exists under other conditions. A similar classification was made for TtDHOase (PDB ID: 2Z00), whose loop conformation closely matched that of ligand-bound BaDHOase (Figure 7B). This ligand-free structure was thus categorized as loop-in.

In contrast, the loop conformation of SaDHOase (PDB ID: 3GRI), despite having a similar loop length to BaDHOase, exhibited a markedly different spatial orientation (Figure 7C). The loop residue Gly151 was positioned approximately 6.9 Å away from the ligand-binding site, making direct interaction unlikely. Therefore, SaDHOase was classified as adopting a loop-out conformation. For AaDHOase, comparison between the NCA-bound (PDB ID: 4BJH) and ligand-free (PDB ID: 1XRF) forms revealed high structural similarity in the loop conformation, including the conserved positioning of the key glycine residue (Gly148) (Figure 7D). Consequently, the unbound AaDHOase structure was also categorized as loop-in. Accordingly, these findings suggest that many type I DHOases, even in the absence of ligands, adopt loop conformations highly similar to ligand-bound loop-in states. This structural predisposition implies that the loop-in conformation may represent a default or energetically favorable state in type I DHOases. Nevertheless, exceptions such as SaDHOase (Figure 7C) highlight that loop dynamics within this class of enzymes can vary. The implications of these predispositions, particularly regarding ligand accessibility, catalysis, and potential for drug targeting, require further investigation through crystallographic studies and molecular dynamics simulations. For example, the complex structure of SaDHOase has not yet been obtained, and acquiring it would be highly valuable for determining whether the short loop in this enzyme is indeed dynamic.

### 2.7. Species-Dependent Loop Conformation Preferences in DHOase

To further explore loop diversity in DHOase, we examined 95 crystal structures (153 monomers) from type I (6 species), type II (7 species), and type III (1 species) enzymes in the PDB. This analysis aimed to determine whether factors such as loop length, amino acid composition, ligand binding status, and type classification influence the adoption of loop-in or loop-out conformations. Overall, 86 monomers were classified as adopting the loop-in conformation, while 67 monomers exhibited loop-out conformations. Type II DHOases exhibited the highest structural variability. While *S. cerevisiae* DHOase consistently adopted the loop-in conformation across all 40 monomers analyzed, *E. coli* DHOase displayed both loop-in and loop-out states. Interestingly, *S. cerevisiae* has the longest active site loop (16 residues), yet loop length alone does not determine conformation; for example, *C. jejuni* DHOase also contains a 16-residue loop but adopts a loop-out state. In type I DHOases, loop-in conformations predominated despite their shorter loops, suggesting a default structural preference. For example, *A. aeolicus* adopted loop-in in all cases, while *S. aureus* displayed only loop-out conformations. In type III (human) DHOase, loop-out states dominated in apo or inhibitor-bound forms, while loop-in appeared in substrate-bound structures. Overall, these findings suggest that loop conformation is influenced by both intrinsic enzyme features and ligand context, with type II and III enzymes showing greater loop plasticity. This variability highlights the importance of loop state selection in docking-based drug design for DHOases.

### 2.8. Ligand-Bound State Analysis and Its Association with Loop Conformations Across DHOase Types

**Figure 8:**
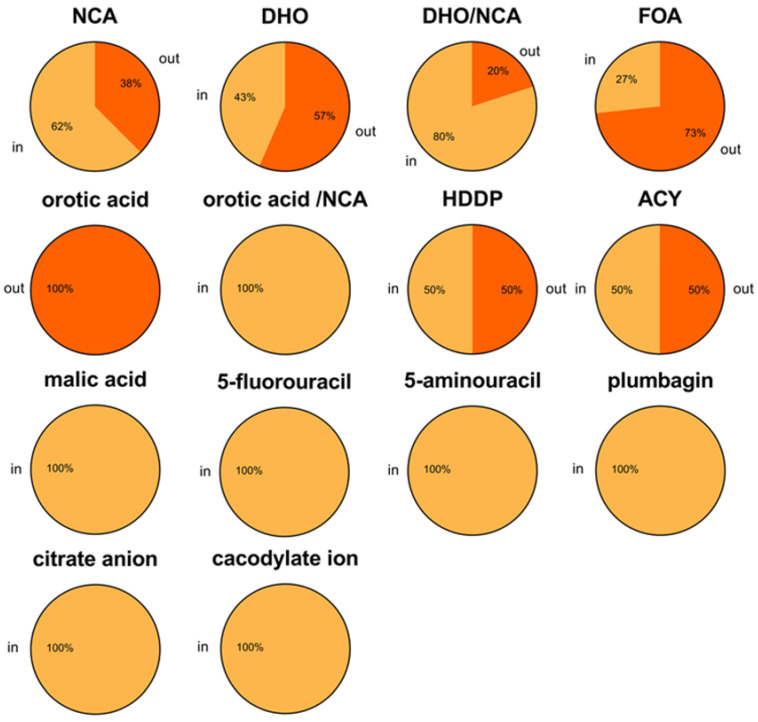
Distribution of active site loop conformations (loop-in vs. loop-out) across various ligands bound to DHOase. Pie charts represent the percentage of loop-in (light orange) and loop-out (dark orange) conformations observed for DHOase structures complexed with different ligands, based on structural data from PDB. Ligands include substrates, products, inhibitors, and small molecules: NCA, DHO, a mixture of DHO/NCA, 5-FOA, orotic acid, HDDP, acetic acid (ACY), malic acid, 5-fluorouracil, 5-aminouracil, plumbagin, citrate anion, and cacodylate ion. Percentages within each chart indicate the proportion of DHOase monomers adopting either loop-in or loop-out conformations for a given ligand. Ligands such as malic acid, 5-fluorouracil, 5-aminouracil, plumbagin, citrate, and cacodylate consistently stabilize the loop-in conformation, while 5-FOA predominantly stabilizes the loop-out state. This analysis highlights ligand-specific preferences in loop dynamics across DHOase structures.

To further understand the relationship between ligand binding and the conformational state of the active site loop in DHOases, we analyzed 117 ligand-bound structures extracted from 95 DHOase entries in PDB (Figure 8 and Table 4). The ligands included substrates, products, inhibitors, and various small molecules, allowing us to assess whether ligand type influences the adoption of loop-in or loop-out conformations. Across the analyzed structures, a total of 81 monomers exhibited the loop-in conformation upon ligand binding, while 36 monomers displayed the loop-out state. This suggests that ligand binding alone does not universally dictate loop conformation, and other factors, such as ligand identity and DHOase type, play crucial roles. For structures bound to the substrate NCA, 10 monomers adopted the loop-in conformation, distributed across all three DHOase types (type I: 2, type II: 3, type III: 5). However, 6 monomers (all type II) with NCA still exhibited the loop-out state, indicating that even substrate binding does not always enforce a loop-in conformation. In the case of the product DHO, 10 monomers (type I: 1, type III: 9) displayed the loop-in conformation, while 13 monomers (type II: 9, type III: 4) showed the loop-out conformation. This diversity indicates the complexity of loop behavior even in substrate/product-bound states. Additionally, some structures contained electron densities corresponding to a mixture of DHO and NCA (annotated as DHO/NCA), which were predominantly associated with the loop-in conformation (8 monomers, all type III), though two structures (type II and III) exhibited loop-out conformations.

For the product-like inhibitor 5-FOA, among 15 5-FOA-bound monomers analyzed, 4 monomers (all from *S. cerevisiae* DHOase) adopted the loop-in conformation, while the remaining 11 monomers (type II: 3, type III: 8) exhibited the loop-out state. This distribution aligns with previous observations in human DHOase, where 5-FOA tends to stabilize the loop-out conformation, mimicking the product release phase [14]. Conversely, in *S. cerevisiae* DHOase [40], 5-FOA binding consistently maintained the loop-in state (Figure 3). For other small-molecule ligands, including 5-fluorouracil (5 monomers: type II: 4, type III: 1), 5-aminouracil (4 monomers, all *S. cerevisiae* DHOase), and plumbagin (4 monomers, all *S. cerevisiae* DHOase), the loop-in conformation was exclusively observed. This finding suggests that these inhibitors preferentially stabilize the loop-in state, particularly in yeast DHOase. Additional small-molecule ligands, including orotic acid, malic acid, acetic acid, and other ions, further illustrate the diversity of loop conformational responses. Notably, malic acid-bound structures (30 monomers; type II: 29, type III: 1) consistently exhibited the loop-in conformation, demonstrating that certain ligands strongly favor loop closure.

### 2.9. pH Conditions Do Not Alter the Active Site Loop Conformation of DHOase

We also investigated whether pH conditions could influence the loop conformation of DHOase. Given that the catalytic reaction of DHOase [31] is reversible and pH-dependent, favoring the conversion of NCA to DHO at pH < 6 and the reverse reaction (DHO to NCA) at pH > 7, the active site loop might be expected to adopt different conformations under varying pH conditions to accommodate these opposing catalytic directions. To evaluate this, we examined crystal structures of human DHOase bound to substrate (DHO/NCA) at pH 5.5 (PDB ID: 4C6E; structure no. 31 in Table 1) and at pH 7.5 (PDB ID: 4C6J; structure no. 34 in Table 1). Both structures consistently exhibited the loop-in conformation. Similarly, for 5-FOA-bound human DHOase, structures at pH 6.0 (PDB ID: 4C6L; structure no. 36 in Table 1) and pH 7.0 (PDB ID: 4C6M; structure no. 37 in Table 1) consistently showed the loop-out conformation. These observations suggest that pH does not significantly influence the loop conformation, even though pH critically affects the direction of the enzymatic reaction. A similar trend was observed for *S. cerevisiae* DHOase. Across structures resolved at pH 6–9 (structure nos. 71–75 in Table 1), all malate-bound forms consistently exhibited the loop-in conformation. This further supports the conclusion that variations in the acid-base environment do not play a decisive role in determining the loop conformation of DHOase.

### 2.10. AlphaFold 3.0 Predictions Reveal Discrepancies Between Predicted and Experimental Loop Conformations in DHOases

**Figure 9:**
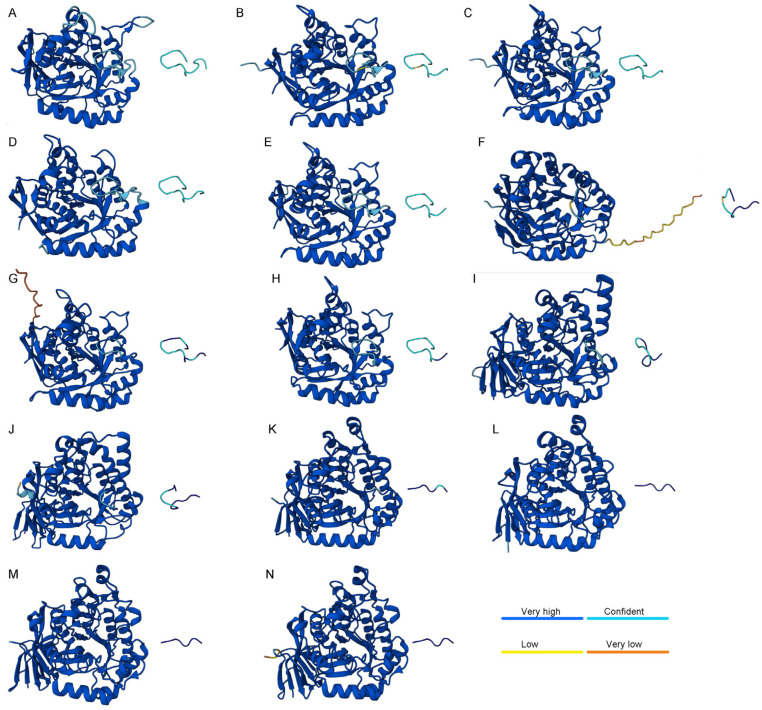
AlphaFold 3.0 structural predictions of DHOases from different species, highlighting active site loop conformations. Predicted structures of DHOases were generated using AlphaFold 3.0 for the following species: (**A**) *S. cerevisiae*, (**B**) *E. coli*, (**C**) *S. enterica*, (**D**) *C. jejuni*, (**E**) *Y. pestis*, (**F**) human DHOase domain, (**G**) *B. cenocepacia*, (**H**) *V. cholerae*, (**I**) *P. gingivalis*, (**J**) *M. jannaschii*, (**K**) *T. thermophilus*, (**L**) *A. aeolicus*, (**M**) *S. aureus*, and (**N**) *B. anthracis*. The flexible active site loop in each structure is shown separately beside the core protein for clarity. Prediction confidence, as assigned by AlphaFold, is indicated by color: blue (very high confidence, pLDDT > 90), light blue (confident, 70 < pLDDT ≤ 90), yellow (low confidence, 50 < pLDDT ≤ 70), and orange (very low confidence, pLDDT ≤ 50). In most structures, the active site loops were predicted with high confidence (blue or light blue), except for *B. cenocepacia* and human DHOases, where terminal disordered regions exhibit lower confidence (yellow or orange). Despite differences in loop sequence and length across species, type II DHOases consistently adopt the loop-in conformation in these models, including *E. coli* DHOase, which predominantly exhibits the loop-out conformation in crystal structures.

Crystal structures of various DHOases indicate that some enzymes, such as *E. coli* and human DHOases, can adopt both loop-in and loop-out conformations, whereas *S. cerevisiae* DHOase consistently exhibits only the loop-in state (Table 2). This raises an intriguing question regarding which loop conformation is preferred according to computational predictions by AI-based tools such as AlphaFold 3.0. To explore this, we employed AlphaFold 3.0 to generate structural models of DHOases from multiple species (Figure 9) and assessed whether these models could reliably predict the flexible active site loop conformations. AlphaFold’s AI-driven predictions are widely recognized for their high accuracy, recently acknowledged with the 2024 Nobel Prize in Chemistry [80,81,82,83]. For this study, we predicted the structures of DHOases from all species with available crystal structures in PDB, including *S. cerevisiae* (Figure 9A), *E. coli* (Figure 9B), *S. enterica* (Figure 9C), *C. jejuni* (Figure 9D), *Y. pestis* (Figure 9E), human (Figure 9F), *B. cenocepacia* (Figure 9G), *V. cholerae* (Figure 9H), *P. gingivalis* (Figure 9I), *M. jannaschii* (Figure 9J), *T. thermophilus* (Figure 9K), *A. aeolicus* (Figure 9L), *S. aureus* (Figure 9M), and *B. anthracis* (Figure 9N). The predicted loop conformations were then compared with their respective experimentally determined crystal structures. Prediction confidence was visualized using AlphaFold’s pLDDT score: blue (very high confidence, pLDDT > 90), light blue (confident, 70 < pLDDT ≤ 90), yellow (low, 50 < pLDDT ≤ 70), and orange (very low, pLDDT ≤ 50). Nearly all predicted models exhibited very high or confident accuracy scores throughout the structure, except for terminal regions in human DHOase (Figure 9F) and *B. cenocepacia* DHOase (Figure 9G), which displayed lower prediction confidence (yellow or orange) but not at the active site loop. Interestingly, despite significant differences in loop sequence composition among species (Table 2), all type II DHOases were predicted to adopt the loop-in conformation, including *E. coli* DHOase (Figure 9B), whose crystal structures overwhelmingly favor the loop-out state (Table 3). This inconsistency between the crystal structures and AlphaFold’s predictions may arise from loop discontinuity or disorder observed in crystallographic models, potentially leading to classification as loop-out despite the intrinsic tendency for loop-in conformations. For human DHOase, which exists in both loop-in and loop-out conformations experimentally, AlphaFold favored the loop-out state. This observation may explain why replacing the human DHOase loop with that from *E. coli* (chimeric loop) renders the enzyme inactive [77], reflecting a functional incompatibility between species-specific loop dynamics.

**Figure 10:**
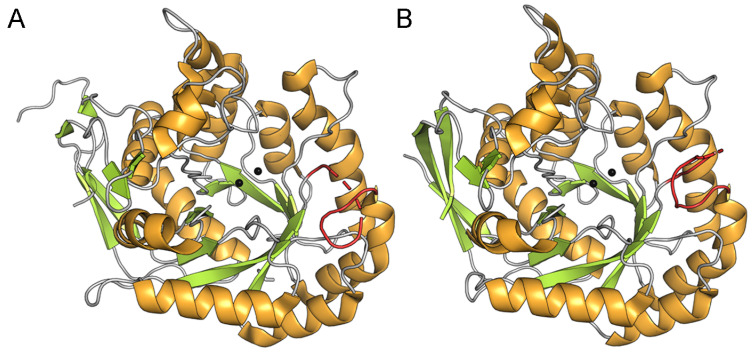
Crystal structures of *B. cenocepacia* DHOase and *V. cholerae* DHOase, highlighting active site loop conformations. Ribbon diagrams of (**A**) *B. cenocepacia* DHOase (PDB ID: 4LFY) and (**B**) *V. cholerae* DHOase (PDB ID: 5VGM) are shown. α-helices are colored in orange, β-strands in green, and loops in gray. The flexible active site loop (loop out) is highlighted in red. Zinc ions within the active site are depicted as black spheres. Both structures exhibit the loop-out conformation, where the active site loop is positioned away from the substrate-binding pocket. This structural observation contrasts with AlphaFold predictions, which suggest a loop-in conformation for these enzymes, underscoring discrepancies between AI-predicted models and experimental crystallographic data. These differences suggest that experimental conditions, ligand occupancy, or intrinsic loop flexibility may influence the observed loop states.

Of particular note are the cases of *B. cenocepacia* (Figure 10A) and *V. cholerae* DHOases (Figure 10B). AlphaFold predicted the active site loops of both enzymes in the loop-in conformation, while their crystal structures consistently display the loop-out state (Table 2). This discrepancy suggests that AlphaFold may overestimate the stability of the loop-in conformation for highly flexible regions, particularly in the absence of ligand-induced stabilization. These mismatches highlight a key limitation of static structure prediction models in accurately capturing the conformational dynamics of flexible loops. Therefore, experimental validation, such as crystallography under varying conditions or molecular dynamics simulations, remains essential for fully understanding loop behavior. Alternatively, it is also possible that the loop-in conformations predicted by AlphaFold for *B. cenocepacia* and *V. cholerae* DHOases do occur but have yet to be captured experimentally, perhaps requiring different crystallization conditions or ligand environments for stabilization.

## 3. Discussion

The de novo pyrimidine biosynthesis pathway is recognized as an attractive target for drug design, particularly against cancer cells, malarial parasites, and other rapidly proliferating pathogens [84,85,86,87,88,89,90,91,92,93,94]. Detailed knowledge of the structures and inhibition mechanisms of enzymes in this pathway, such as DHOase in this study, offers a significant advantage for the development of targeted inhibitors. However, DHOase exhibits dynamic conformational changes at its active site during catalysis, which presents challenges for structure-based drug design [6]. For instance, although 5-FOA is a known inhibitor of *E. coli* [49,75], *P. falciparum* [26,27,28,29], and *S. cerevisiae* DHOases (Figure 2), it cannot be docked into DHOase structures that adopt the loop-in conformation (Figure 6). This study presents an integrative analysis of the conformational dynamics of the active site loop in DHOase (Table 1), with a focus on the structural and functional implications of the loop-in and loop-out states across different DHOase types. By combining experimental inhibition assays, structural comparisons, docking simulations, and AI-driven predictions, we offer new insights into the conformational plasticity of the DHOase loop and its potential as a therapeutic target.

The identification of 5-FOA and myricetin as inhibitors of *S. cerevisiae* DHOase (Figure 2) further expands our understanding of inhibitor specificity across DHOase types. These compounds possess markedly different chemical structures, which may account for their distinct inhibitory effects. Notably, myricetin exhibited stronger inhibition (IC_50_ = 12.48 ± 0.47 μM) compared to 5-FOA against *S. cerevisiae* DHOase. This suggests that flavonol-based inhibitors [95,96,97,98,99], such as myricetin [100,101,102,103,104,105,106], may serve as more potent scaffolds for selectively targeting yeast DHOase. In addition to inhibiting DHOase, myricetin is known to inhibit bacterial helicase DnaB [107], PriA [108], dihydropyrimidinase (DHPase) [109], single-stranded DNA-binding protein [110,111,112], and the SARS-CoV-2 3CL protease [113]. Since DHPase [114,115,116,117,118,119] and DHOase both belong to the cyclic amidohydrolase family [116,120,121], myricetin may serve as a lead compound or “dirty drug,” capable of targeting multiple enzymes within this family, including hydantoinase [122,123] and allantoinase (ALLase) [12,124,125]. Given the catalytic importance of the dynamic loop in DHPase, ALLase, and DHOase, this study reveals the need to carefully consider loop conformation when performing docking experiments for drug screening. Specifically, using loop-in conformations as docking templates may lead to inaccurate predictions, as demonstrated in this study (Figure 4, Figure 5 and Figure 6). Therefore, accurately assessing loop dynamics is crucial for the effective design and development of inhibitors targeting DHOase, as well as DHPase and ALLase.

**Figure 11:**
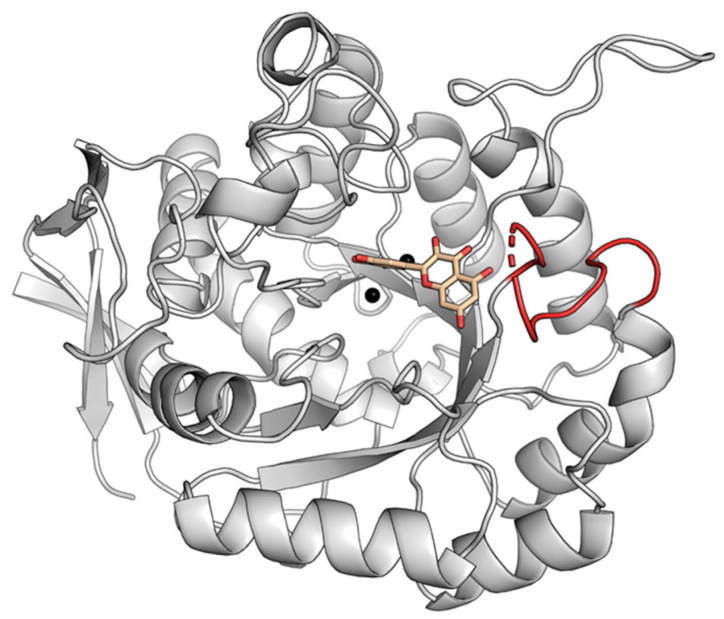
Docking result of myricetin to the loop-deleted (residues 104–108) *S. cerevisiae* DHOase. To mimic the loop-out conformation, residues 104–108 of the dynamic loop in *S. cerevisiae* DHOase were manually deleted prior to the docking experiment. The top-ranked binding pose showed successful docking into the active site with an affinity of –8.4 kcal/mol, suggesting that the loop-in conformation is unsuitable as a docking template.

Our docking studies further highlighted the practical challenges of modeling ligand interactions with DHOase. In *S. cerevisiae* DHOase, the loop-in conformation sterically hindered the docking of both 5-FOA and myricetin, despite crystallographic data confirming the binding of 5-FOA at the active site [40]. This limitation indicates the importance of selecting appropriate structural templates—specifically favoring loop-out conformations in docking simulations to permit ligand access. If residues 104–108 on the dynamic loop of *S. cerevisiae* DHOase are deleted (to mimic the loop-out conformation), the top-ranked binding pose showed successful docking into the active site with an affinity of –8.4 kcal/mol, suggesting that the loop-in conformation is unsuitable as a docking template (Figure 11). This was consistently observed across multiple species, where successful docking was only achieved with loop-out conformations, irrespective of enzyme type (Table 1, Table 2 and Table 3). However, although the loop-in state impedes docking, it plays a critical role in stabilizing inhibitor interactions after binding, as demonstrated in the *S. cerevisiae* DHOase complex [39,40,41,42]. Therefore, targeting the loop-in state through post-docking loop repositioning strategies may be an effective approach for therapeutic applications in such cases. In addition, our structural analysis reinforced this concept by revealing distinct 5-FOA binding modes across different DHOase types. The consistent loop-in conformation observed in *S. cerevisiae* DHOase contrasts with the loop-out conformation seen in human and *E. coli* DHOases, highlighting the loop’s role in modulating ligand binding. This conformational divergence provides further evidence challenging the classical model derived from *E. coli* DHOase, in which the loop-in state is associated with substrate binding and the loop-out state with product release or non-substrate/product-like ligand binding. These findings suggest that loop conformation is not universally ligand-dependent but varies significantly across DHOase types and species (Table 4).

Expanding our analysis to 95 crystal structures comprising 153 monomers, we systematically categorized loop conformations across different DHOase types (Table 1). Type I DHOases predominantly adopted loop-in conformations (Figure 7), even in the absence of ligands, whereas type II and III enzymes exhibited significant loop plasticity, with both loop-in and loop-out states observed (Table 2 and Table 3). This variability was most pronounced in type II enzymes, reflecting their adaptive binding roles and potential evolutionary divergence. Notably, the consistent loop-in conformation of *S. cerevisiae* DHOase, in contrast to the mixed loop states observed in *E. coli* DHOase, may highlight the influence of enzyme organization. Yeast DHOase operates as part of a CAD-like multifunctional complex [43,45,46], whereas bacterial DHOases function independently alongside separate partner proteins [15,16,32,48,49], suggesting distinct evolutionary pressures shaping loop behavior. However, further investigations are needed to elucidate the mechanistic role of this dynamic loop in catalysis and regulation. Additionally, our ligand-binding analysis provided further evidence that loop conformation is influenced more by ligand type than by ligand presence alone. Small molecule ligands such as malic acid consistently favored loop-in conformations, whereas inhibitors like 5-FOA stabilized different loop states depending on the DHOase species (Figure 8). These findings suggest that loop dynamics are highly context-dependent, modulated by both the intrinsic structural properties of the enzyme and the chemical nature of the ligand.

Compared to human DHOase, which has been more extensively studied biochemically, the DHOases from *M. jannaschii* and *P. gingivalis*, particularly their interactions with partner proteins, remain poorly characterized. Therefore, in this study, we continue to classify these longer DHOases (comprising more than 400 amino acid residues) as type I, consistent with earlier classification systems. Although a structural model for *P. gingivalis* DHOase is available in the PDB, it has not yet been described in a peer-reviewed publication. This underscores the need for further biochemical and structural investigations to clarify its potential functions.

**Figure 12:**
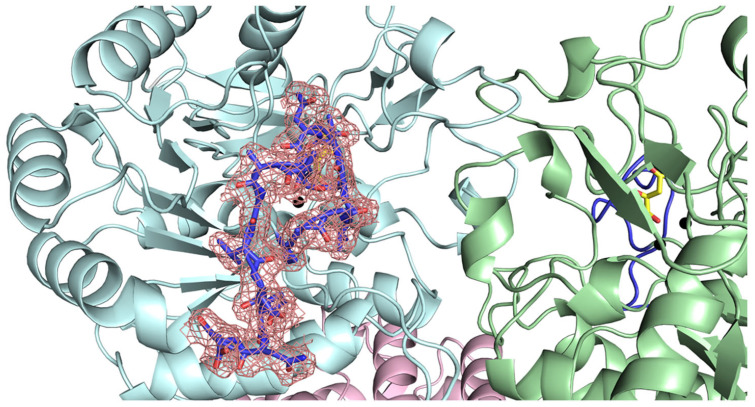
The tetrameric structure of *S. cerevisiae* DHOase. The loop (colored in dark blue) does not interact with another *S. cerevisiae* DHOase monomer. This indicates that the loop conformation is not caused by crystal packing or monomer–monomer interactions.

Whether *S. cerevisiae* DHOase can adopt a loop-out conformation for ligand binding remains unknown, as its apo form has not yet been structurally elucidated. Based on the analysis of intersubunit interactions, the loop-in conformation may be not caused by crystal packing, as the loop does not interact with another *S. cerevisiae* DHOase monomer (Figure 12). Given that the flexible loop in *S. cerevisiae* DHOase is the longest among the analyzed DHOases, it may exhibit unique binding mechanisms (Figure 3). It is possible that conformational cycling of the loop between loop-in and loop-out states, as observed in other DHOases, is not required in *S. cerevisiae* due to steric hindrance imposed by the longer loop. Whether these differences in binding modes across DHOases are species-specific or influenced by crystallographic conditions requires further experimental investigation. Possibly, loop-swapping experiments to create chimeric *S. cerevisiae* DHOase could serve as an initial approach to evaluate loop compatibility across species. For example, a previously constructed human DHOase chimera bearing the flexible loop from *E. coli* DHOase was inactive, providing strong evidence that the functional roles of these loops differ between species. Since *S. cerevisiae* DHOase is neither covalently linked to a partner protein, as in human DHOase within the CAD complex, nor operates entirely independently like bacterial DHOases (Figure 1), it may possess unique structural adaptations acquired through evolution. These features include its unusually long loop and its consistent loop-in conformation across all solved 10 structures (Table 2), though the functional significance of these characteristics remains to be elucidated.

Our AlphaFold 3.0 predictions revealed both concordances and discrepancies with experimental data. Notably, despite differences in loop sequence composition, AlphaFold consistently predicted loop-in conformations for all type II DHOases (Figure 9), including *E. coli*, which predominantly adopts the loop-out state in crystal structures (Table 2). This discrepancy may highlight the limitations of static AI-based predictions for flexible regions like active site loops and emphasizes the need for experimental validation and dynamic modeling. Interestingly, for *B. cenocepacia* and *V. cholerae* DHOases (Figure 10), AlphaFold predicted loop-in conformations (Supplementary Materials Figure S3), whereas the experimentally determined structures displayed loop-out states. These findings suggest that alternative loop-in conformations may exist but have yet to be captured crystallographically.

For Type I DHOases, given their very short loop, we acknowledge that interspecies comparisons must be interpreted with caution. Although we classified different conformations, such as loop-in for *A. aeolicus* and *B. anthracis* DHOases and loop-out for *S. aureus* DHOase, the loops in these enzymes appear to adopt a similar conformation. This is likely due to their limited length, which restricts significant movement. Nevertheless, differences still exist in the architecture of the active site. Variations in the number of zinc ions and the coordination geometry may also influence loop positioning. For instance, in PDB ID 3GRI (*S. aureus* DHOase), only a single Zn ion is present, and the key catalytic residue Asp150 is positioned far from the metal center. In contrast, in PDB ID 4YIW (*B. anthracis* DHOase), two Zn ions are coordinated by Asp151, forming a bridge between the metals. These structural distinctions may reflect species-specific adaptations or differences in crystallization conditions. Notably, in Type I DHOases such as that from *A. aeolicus*, structural studies have shown that the flexible loop makes minimal direct contact with the substrate NCA [64]. This suggests that additional interactions, such as with ATCase, may be necessary for proper substrate positioning and catalysis. These findings support the idea that loop conformational dynamics in Type I DHOases may be regulated by higher-order protein–protein interactions rather than by ligand binding alone.

**Figure 13:**
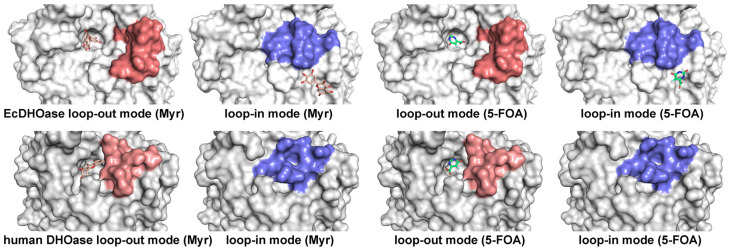
Docking results of myricetin and 5-FOA to EcDHOase and human DHOase. Docking experiments with 5-FOA and myricetin confirmed that the loop-in conformation prevented productive active-site docking.

In conclusion, the observed variability in loop states across DHOase types challenges the classical catalytic model based on loop-in and loop-out transitions (Figure 13) and provides valuable insights for the development of selective inhibitors, particularly when targeting the loop-in state, as exemplified by myricetin’s inhibition of *S. cerevisiae* DHOase.

## 4. Materials and Methods

### 4.1. Chemicals and Bacterial Strain

All chemicals, including 5-FOA and myricetin, were of the highest analytical grade and purchased from Sigma-Aldrich (St. Louis, MO, USA). The *E. coli* strain BL21(DE3) pLysS (Novagen, Worcestershire, UK) was used for recombinant DHOase expression.

### 4.2. Expression and Purification of the Recombinant Protein

The plasmid construction for *S. cerevisiae* DHOase expression was previously reported [126]. Recombinant protein purification followed established protocols [127]. Briefly, *E. coli* BL21(DE3) cells were transformed with the expression plasmid, and protein expression was induced with 1 mM isopropyl β-D-1-thiogalactopyranoside (IPTG). The recombinant protein was purified from the soluble fraction using Ni^2+^-affinity chromatography (HiTrap HP; GE Healthcare Bio-Sciences, Uppsala, Sweden) and eluted with buffer A (20 mM Tris–HCl, 250 mM imidazole, 0.5 M NaCl, pH 7.9). The eluate was dialyzed against buffer B (20 mM Tris–HCl, 0.1 M NaCl, pH 7.9). Protein purity exceeded 97%, as verified by SDS–PAGE (Mini-PROTEAN Tetra System; Bio-Rad, Hercules, CA, USA).

### 4.3. Enzyme Assay

A rapid spectrophotometric assay was employed to measure the activity of *S. cerevisiae* DHOase [128]. The hydrolysis of DHO was monitored at 25 °C by measuring the decrease in absorbance at 230 nm. The reaction mixture (2 mL) contained 0.5 mM DHO and 100 mM Tris–HCl (pH 8.0). The extinction coefficient of DHO was 0.92 mM^−1^·cm^−1^ at 230 nm. The reaction was initiated by adding the purified enzyme, and absorbance changes were recorded using a UV/Vis spectrophotometer (Hitachi U-3300; Hitachi High-Technologies, Tokyo, Japan).

### 4.4. Binding Analysis Using AutoDock Vina

The interactions between various compounds and different DHOases (Supplementary Materials Table S1) were analyzed using AutoDock Vina (Version 2.0) [129,130,131]. DHOase structures were obtained from PDB database. Pre-docking preparations, including charge assignments and grid box settings, were performed using AutoDockTools (v1.5.6). Ligand 2D structures were retrieved from PubChem and converted to .sdf format. Ligands and protein targets were then prepared as PDBQT files for docking using AutoDock Vina via the PyRx Virtual Screening Tool (v1.1). Docking results were visualized and analyzed using PyMOL v2.2.0.

### 4.5. Collection and Analysis of DHOase Structures from PDB for Active Site Loop Conformation

All available DHOase structures from different species deposited in PDB were systematically collected and manually analyzed for active site loop conformations. Loop states were classified based on ligand interaction: if the loop residues directly interacted with the ligand at the active site, the conformation was categorized as loop-in; if no interaction occurred between the loop and the bound ligand, the conformation was designated as loop-out.

## Figures and Tables

**Table 1:** Summary of the 97 dihydroorotase structures available in the Protein Data Bank (PDB).

No.	PDB ID	Structure	Unique Ligands	Amino Acid Residues	Seq. Id.	TM-Score	RMSD	Type	Loop State in the Monomer
1	1J79	*Escherichia coli* dihydroorotase	Zn(αβ), NCA orotic acid	1–348	100	1	0	II	A: out B: out *^a^*
2	1XGE	*Escherichia coli* dihydroorotase	Zn(αβ), DHO, NCA	1–348	98.2	0.999	0.18	II	A: out B: in
3	1XRF	*Aquifex aeolicus* dihydroorotase	Zn(α)	1–422	16.1	0.722	2.79	I	A: in *^b^*
4	1XRT	*Aquifex aeolicus* dihydroorotase	Zn(α)	1–422	15.9	0.723	2.91	I	A: in *^b^* B: in *^b^*
5	2E25	The T109S mutant of *Escherichia coli* dihydroorotase in complex with FOA	Zn(αβ), FOA	1–348	99.1	0.998	0.28	II	A: out
6	2EG6	*Escherichia coli* dihydroorotase	Zn(αβ)	1–348	99.4	0.999	0.2	II	A: out B: out *^a^*
7	2EG7	*Escherichia coli* dihydroorotase in complex with HDDP	Zn(αβ), HDDP	1–348	99.4	0.999	0.21	II	A: out B: in
8	2EG8	*Escherichia coli* dihydroorotase in complex with FOA	Zn(αβ), FOA	1–348	99.4	0.999	0.22	II	A: out B: out *^a^*
9	2GWN	*Porphyromonas gingivalis* dihydroorotase	Zn(αβ), cacodylate ion	1–449	14.1	0.846	2.49	I	A: in
10 *	2OGJ	*Agrobacterium fabrum* dihydroorotase	Zn(αβ) imidazole	1–407	ND	0.609	3.93	ND	ND
11	2Z00	*Thermus thermophilus* dihydroorotase	Zn(αβ)	1–426	16.9	0.851	2.59	I	A: in *^b^*
12	2Z24	Thr110Ser dihydroorotase from *Escherichia coli*	Zn(αβ), DHO, NCA	1–348	99.1	0.999	0.21	II	A: out B: out *^a^*
13	2Z25	Thr110Val dihydroorotase from *Escherichia coli*	Zn(αβ), DHO, NCA	1–348	99.1	0.999	0.21	II	A: out B: in
14	2Z26	Thr110Ala dihydroorotase from *Escherichia coli*	Zn(αβ), mixed NCA/DHO, NCA	1–348	99.1	0.999	0.23	II	A: out B: out *^a^*
15	2Z27	Thr109Ser dihydroorotase from *Escherichia coli*	Zn(αβ), DHO, NCA	1–348	99.1	0.999	0.22	II	A: out B: out *^a^*
16	2Z28	Thr109Val dihydroorotase from *Escherichia coli*	Zn(αβ), DHO, NCA	1–348	99.1	0.999	0.21	II	A: out B: out *^a^*
17	2Z29	Thr109Ala dihydroorotase from *Escherichia coli*	Zn(αβ), DHO, NCA	1–348	99.1	0.999	0.22	II	A: out B: out *^a^*
18	2Z2A	Thr109Gly dihydroorotase from *Escherichia coli*	Zn(αβ), NCA, mixed NCA/DHO	1–348	99.1	0.999	0.22	II	A: out B: out *^a^*
19	2Z2B	Deletion 107–116 mutant of dihydroorotase from *E. coli*	Zn(αβ)	1–338	96.1	0.960	0.51	II	A: out
20	3D6N	The *Aquifex aeolicus* dihydroorotase complex	Zn(α), citrate anion	1–422	16.7	0.840	2.75	I	A: in
21	3GRI	*Staphylococcus aureus* dihydroorotase	Zn(α)	1–424	15.1	0.829	2.69	I	A: out B: out
22	3JZE	Dihydroorotase from *Salmonella enterica* subsp. *enterica* serovar Typhimurium str. LT2	Zn(αβ), ACY	1–348	87.5	0.980	1.3	II	A: in B: out C: in D: out
23	3MJM	His257Ala dihydroorotase from *Escherichia coli*	Zn(αβ), DHO, NCA	1–348	97.9	0.999	0.2	II	A: out B: in
24	3MPG	*Bacillus anthracis* dihydroorotase	Zn(αβ)	1–428	16.6	0.840	2.80	I	A: in *^b^* B: in *^b^*
25	3PNU	*Campylobacter jejuni* dihydroorotase	Zn(αβ)	1–335	36.2	0.903	1.90	II	A: out B: out
26	4BJH	The *Aquifex aeolicus* dihydroorotase (H180A, H232A) complex	Zn(α), DHO	1–422	16.1	0.841	2.75	I	A: in
27	4BY3	Human dihydroorotase in apo-form obtained recombinantly from *E. coli.*	Zn(αβγ)	1456–1822	15.7	0.818	2.94	III	A: out
28	4C6B	Human dihydroorotase with incomplete active site, obtained recombinantly from *E. coli*	Without metal	1456–1822	15.4	0.785	2.70	III	A: out
29	4C6C	Human dihydroorotase obtained recombinantly from HEK293 cells	Zn(αβγ)	1456–1822	15.8	0.818	2.64	III	A: out
30	4C6D	Human dihydroorotase bound to substrate at pH 6.0	Zn(αβγ), mixed DHO/NCA	1456–1822	15.7	0.814	2.64	III	A: in/out
31	4C6E	Human dihydroorotase bound to substrate at pH 5.5	Zn(αβ), mixed DHO/NCA	1456–1822	15.5	0.814	2.64	III	A: in/out
32	4C6F	Human dihydroorotase bound to substrate at pH 6.5	Zn(αβγ), mixed DHO/NCA	1456–1822	15.7	0.814	2.64	III	A: in/out
33	4C6I	Human dihydroorotase bound to substrate at pH 7.0	Zn(αβγ), mixed DHO/NCA	1456–1822	15.7	0.813	2.65	III	A: in/out
34	4C6J	Human dihydroorotase bound to substrate at pH 7.5	Zn(αβγ), mixed DHO/NCA	1456–1822	15.7	0.813	2.65	III	A: in/out
35	4C6K	Human dihydroorotase bound to substrate at pH 8.0	Zn(αβγ), mixed DHO/NCA	1456–1822	15.5	0.814	2.64	III	A: in/out
36	4C6L	Human dihydroorotase bound to the inhibitor fluoroorotate at pH 6.0	Zn(αβ), FOA	1456–1822	15.5	0.819	2.64	III	A: out
37	4C6M	Human dihydroorotase bound to the inhibitor fluoroorotate at pH 7.0	Zn(αβγ), FOA	1456–1822	15.5	0.819	2.65	III	A: out
38	4C6N	Human dihydroorotase E1637T mutant bound to substrate at pH 6.0	Zn(αβ), NCA	1456–1822	15.4	0.812	2.63	III	A: in
39	4C6O	Human dihydroorotase C1613S mutant in apo-form at pH 6.0	Zn(αβ)	1456–1822	15.7	0.818	2.64	III	A: out
40	4C6P	Human dihydroorotase C1613S mutant in apo-form at pH 7.0	Zn(αβγ)	1456–1822	15.5	0.819	2.64	III	A: out
41	4C6Q	Human dihydroorotase C1613S mutant bound to substrate at pH 7.0	Zn(αβ), mixed orotic acid/ NCA	1456–1822	15.5	0.814	2.64	III	A: in/out
42	4LFY	*Burkholderia cenocepacia* dihydroorotase	Zn(αβ)	1–364	53.9	0.969	0.94	II	A: out *^a^* B: out *^a^*
43	4YIW	*Bacillus anthracis* dihydroorotase	Zn(αβ), NCA	1–428	16.6	0.842	2.78	I	A: in B: in
44 #	5NNL	Inactive dihydroorotase-like domain of *Chaetomium thermophilum* CAD	Without metal	1519–1855	10	0.747	3.26	ND	ND
45	5VGM	*Vibrio cholerae* dihydroorotase	Zn(αβ)	1–342	53	0.939	0.94	II	A: out *^a^* B: out *^a^*
46	5YNZ	Human dihydroorotase K1556A mutant	Zn(α)	1456–1822	15.2	0.819	2.65	III	A: out
47	6CTY	*Yersinia pestis* dihydroorotase	Zn(αβ), malic acid	1–348	71.1	0.971	1.32	II	A: in B: in C: in D: in E: out *^a^* F: in
48	6GDD	Dihydroorotase from *Aquifex aeolicus* under 1200 bar of hydrostatic pressure	Zn(α)	1–422	16.0	0.744	2.91	I	A: in *^b^*
49	6GDE	Dihydroorotase from *Aquifex aeolicus* under 600 bar of hydrostatic pressure	Zn(α)	422	16.1	0.727	2.63	I	A: in *^b^*
50	6GDF	Dihydroorotase from *Aquifex aeolicus* standard (P,T)	Zn(α)	1–422	16.1	0.734	2.79	I	A: in *^b^*
51	6HFD	Human dihydroorotase mutant F1563L apo structure	Zn(αβγ)	1456–1822	15.7	0.818	2.65	III	A: out
52	6HFE	Human dihydroorotase mutant F1563T apo structure	Zn(αβγ)	1456–1822	15.7	0.819	2.63	III	A: out
53	6HFF	Human dihydroorotase mutant F1563Y apo structure	Zn(αβγ)	1456–1822	15.7	0.818	2.64	III	A: out
54	6HFH	Human dihydroorotase mutant F1563A co-crystallized with carbamoyl aspartate at pH 7.0	Zn(αβγ), DHO *^c^*	1456–1822	15.7	0.818	2.65	III	A: out
55	6HFI	Human dihydroorotase mutant F1563A apo structure	Zn(αβγ)	1456–1822	15.7	0.818	2.65	III	A: out
56	6HFJ	Human dihydroorotase mutant F1563A co-crystallized with carbamoyl aspartate at pH 7.5	Zn(αβγ), DHO *^c^*	1456–1822	16.1	0.818	2.66	III	A: out
57	6HFK	Human dihydroorotase mutant F1563L co-crystallized with carbamoyl aspartate at pH 6.5	Zn(αβγ), DHO *^c^*	1456–1822	15.5	0.818	2.66	III	A: out
58	6HFL	Human dihydroorotase mutant F1563L co-crystallized with carbamoyl aspartate at pH 7.0	Zn(αβγ), DHO *^c^*	1456–1822	15.7	0.818	2.65	III	A: out
59	6HFN	Human dihydroorotase mutant F1563L co-crystallized with carbamoyl aspartate at pH 7.5	Zn(αβγ), DHO *^c^*	1456–1822	16.1	0.818	2.66	III	A: out
60	6HFP	Human dihydroorotase mutant F1563T co-crystallized with carbamoyl aspartate at pH 7.0	Zn(αβγ), DHO *^c^*	1456–1822	15.7	0.819	2.63	III	A: out
61	6HFQ	Human dihydroorotase mutant F1563T co-crystallized with carbamoyl aspartate at pH 7.5	Zn(αβγ), DHO *^c^*	1456–1822	16.1	0.819	2.63	III	A: out
62	6HFR	Human dihydroorotase mutant F1563Y co-crystallized with carbamoyl aspartate at pH 7.0	Zn(αβγ), NCA	1456–1822	15.7	0.813	2.65	III	A: in
63	6HFU	Human dihydroorotase mutant F1563Y co-crystallized with carbamoyl aspartate at pH 7.5	Zn(αβγ), NCA	1456–1822	16.1	0.813	2.65	III	A: in
64	6HFS	Human dihydroorotase mutant F1563Y co-crystallized with carbamoyl aspartate at pH 6.5	Zn(αβγ), NCA	1456–1822	15.5	0.813	2.65	III	A: in
65	6HG1	Hybrid dihydroorotase domain of human CAD with *E. coli* flexible loop in apo state	Zn(αβ)	1456–1822	19.1	0.821	2.78	III	A: out *^a^*
66	6HG2	Hybrid dihydroorotase domain of human CAD with *E. coli* flexible loop, bound to FOA	Zn(αβ), FOA	1456–1822	16.3	0.808	2.52	III	A: out *^a^*
67	6HG3	Hybrid dihydroorotase domain of human CAD with *E. coli* flexible loop, bound to dihydroorotate	Zn(αβ), DHO	1456–1822	16.9	0.815	2.62	III	A: out *^a^*
68	6L0A	*Saccharomyces cerevisiae* dihydroorotase complexed with malate at pH 7	Zn(αβ), malic acid	1–364	28.2	0.926	1.92	II	A: in B: in C: in D: in
69	6L0B	*Saccharomyces cerevisiae* dihydroorotase complexed with 5-fluorouracil	Zn(αβ), 5-fluorouracil	1–364	28.2	0.925	1.97	II	A: in B: in C: in D: in
70	6L0F	*Saccharomyces cerevisiae* dihydroorotase complexed with 5-aminouracil	Zn(αβ), 5-aminouracil	1–364	28.2	0.926	1.96	II	A: in B: in C: in D: in
71	6L0G	*Saccharomyces cerevisiae* dihydroorotase complexed with malate at pH 6	Zn(αβ), malic acid	1–364	28.2	0.926	1.95	II	A: in B: in C: in D: in
72	6L0H	*Saccharomyces cerevisiae* dihydroorotase complexed with malate at pH 7	Zn(αβ), malic acid	1–364	28.2	0.927	1.92	II	A: in B: in C: in D: in
73	6L0I	*Saccharomyces cerevisiae* dihydroorotase complexed with malate at pH 6.5	Zn(αβ), malic acid	1–364	28.2	0.925	1.97	II	A: in B: in C: in D: in
74	6L0J	*Saccharomyces cerevisiae* dihydroorotase complexed with malate at pH 7.5	Zn(αβ), malic acid	1–364	28.2	0.926	1.96	II	A: in B: in C: in D: in
75	6L0K	*Saccharomyces cerevisiae* dihydroorotase complexed with malate at pH 9	Zn(αβ), malic acid	1–364	28.2	0.924	2	II	A: in B: in C: in D: in
76	7CA0	*Saccharomyces cerevisiae* dihydroorotase complexed with 5-fluoroorotic acid	Zn(αβ), FOA	1–364	28.2	0.926	1.95	II	A: in B: in C: in D: in
77	7CA1	*Saccharomyces cerevisiae* dihydroorotase complexed with plumbagin	Zn(αβ), plumbagin	1–364	28.2	0.925	1.98	II	A: in B: in C: in D: in
78	7UOF	*Methanococcus jannaschii* dihydroorotase	Zn(αβ)	1–423	13.5	0.845	2.66	I	A: out
79	8GVZ	Human dihydroorotase in complex with the anticancer drug 5-fluorouracil	Zn(αβ), 5-fluorouracil	1456–1822	15.1	0.813	2.65	III	A: in
80	8GW0	Human dihydroorotase in complex with malic acid	Zn(αβ), malic acid	1456–1822	15.6	0.813	2.65	III	A: in
81	8PBE	Human dihydroorotase mutant K1556T bound to the substrate carbamoyl aspartate	Zn(αβγ), NCA	1456–1822	14.8	0.813	2.67	III	A: in
82	8PBG	Human dihydroorotase mutant K1556T bound to the inhibitor fluoroorotate	Zn(αβγ), FOA	1456–1822	15.2	0.814	2.64	III	A: out
83	8PBH	Human dihydroorotase mutant R1617Q bound to the substrate carbamoyl aspartate	Zn(αβγ), NCA	1456–1822	15.4	0.814	2.63	III	A: in
84	8PBI	Human dihydroorotase mutant R1617Q bound to the inhibitor fluoroorotate	Zn(αβγ), FOA	1456–1822	15.1	0.818	2.64	III	A: out
85	8PBJ	Human dihydroorotase mutant R1722W bound to the substrate carbamoyl aspartate	Zn(αβγ), NCA	1456–1822	15.1	0.814	2.64	III	A: in
86	8PBK	Human dihydroorotase mutant R1722W bound to the inhibitor fluoroorotate	Zn(αβγ), FOA	1456–1822	15.8	0.819	2.64	III	A: out
87	8PBM	Human dihydroorotase mutant R1789Q bound to the substrate dihydroorotate	Zn(αβγ), DHO	1456–1822	15.5	0.813	2.65	III	A: in/out
88	8PBN	Human dihydroorotase mutant R1789Q bound to the inhibitor fluoroorotate	Zn(αβγ), FOA	1456–1822	15.5	0.818	2.65	III	A: out
89	8PBP	Human dihydroorotase mutant R1785C bound to the substrate carbamoyl aspartate	Zn(αβγ), NCA/DHO	1456–1822	15.5	0.814	2.64	III	A: in
90	8PBQ	Human dihydroorotase mutant R1810Q bound to the substrate carbamoyl aspartate	Zn(αβγ), NCA	1456–1822	15.8	0.814	2.64	III	A: in
91	8PBR	Human dihydroorotase mutant R1475Q in apo form	Zn(αβγ)	1456–1822	15.5	0.820	2.66	III	A: out
92	8PBS	Human dihydroorotase mutant K1482M in apo form	Zn(αβγ)	1456–1822	15.7	0.806	2.71	III	A: out
93	8PBT	Human dihydroorotase mutant K1482M bound to the substrate dihydroorotate	Zn(αβγ), DHO	1456–1822	16.1	0.818	2.66	III	A: out
94	8PBU	Human dihydroorotase mutant K1482M bound to the inhibitor fluoroorotate	Zn(αβγ), FOA	1456–1822	16.1	0.818	2.66	III	A: out
95	9FS1	Human dihydroorotase mutant S1538L bound to carbamoyl aspartate	Zn(αβγ), NCA	1460–1821	15.1	0.813	2.65	III	A: in
96	9FS2	Human dihydroorotase mutant S1538A bound to substrate	Zn(αβγ), DHO/NCA	1460–1821	15.8	0.814	2.65	III	A: in/out
97	9FS3	Human dihydroorotase mutant S1538A in apo form	Zn(αβγ)	1460–1821	15.5	0.818	2.65	III	A: out

Loop-in state—The flexible loop adopts an inward orientation, positioning key residues to interact directly with the ligand. Loop-out state—The flexible loop adopts an outward orientation, in which key residues are positioned away from the ligand and do not participate in direct interactions. Abbreviations: NCA, N-carbamoyl-L-aspartate; DHO, dihydroorotate; FOA, 5-fluoroorotic acid; HDDP, 2-oxo-1,2,3,6-tetrahydropyrimidine-4,6-dicarboxylate; ACY, acetic acid. *^a^* Some residues in the loop were not determined, possibly due to structural disorder. *^b^* Although no ligand is bound, the loop conformation resembles the ligand-bound state and is therefore classified as loop-in. *^c^* The ligand is indicated as dihydroorotate (DHO). * This protein may not be a bona fide DHOase. # This protein is structurally similar to DHOase but lacks catalytic activity.

**Table 2:** Summary of the active site loop conformations in 153 monomers across 95 DHOase structures from the PDB.

Type	DHOase	Loop Length	Loop Composition	In	Out	Note
II	*S. cerevisiae* DHOase	16	PAGVTTNSAAGVDPND	40	0	10 structures; 40 monomers (10 tetramers)
II	*C. jejuni* DHOase	16	PAGITTNSNGGVSSFD	0	2	1 structure; 2 monomers (1 dimer)
II	*E. coli* DHOase	14	PANATTNSSHGVTS	4	24	15 structures; 28 monomers (2 monomers and 13 dimers)
II	*S. enterica* DHOase	14	PANATTNSSHGVTS	2	2	1 structure; 4 monomers (1 tetramer)
II	*Y. pestis* DHOase	14	PANATTNSTHGVSD	5	1	1 structure; 6 monomers (1 hexamer)
II	*B. cenocepacia* DHOase	14	PAGATTNSDHGVTD	0	2	1 structure; 2 monomers (1 dimer)
II	*V. cholera* DHOase	14	PAGATTNSDSGVTS	0	2	1 structure; 2 monomers (1 dimer)
III	Human DHOase	12	LNETFSELRLDS	21	31	52 structures; 52 monomers (52 monomers)
I	*M.* jannaschii DHOase	12	MVKSVGDLFIED	0	1	1 structure; 1 monomer (1 monomer)
I	*P. gingivalis* DHOase	11	LGSSTGNMLVD	1	0	1 structure; 1 monomer (1 monomer)
I	*T. thermophilus* DHOase	6	GRTNED	1	0	1 structure; 1 monomer (1 monomer)
I	*A. aeolicus* DHOase	6	GSPVMD	8	0	7 structures; 8 monomers (6 monomers and 1 dimer)
I	*S. aureus* DHOase	6	GVGVQT	0	2	1 structure; 2 monomers (1 dimer)
I	*B. anthracis* DHOase	6	GVGVQD	4	0	2 structures; 4 monomers (2 dimers)
Total: 6 type I, 7 type II, and 1 type III DHOases		6–16; Typically important residues: G for type I; TT for type II; and TF for type III		86	67	95 structures; 153 monomers (63 monomers, 20 dimers, 11 tetramers, and 1 hexamer)

The listed DHOases are ordered according to loop length (number of amino acid residues).

**Table 3:** Summary of active site loop conformations in 153 monomers from 95 DHOase structures in the PDB, classified by ligand-bound and unbound states.

Type	Loop In (with Ligand)	Loop Out (with Ligand)	Note
I	14 (5)	3 (0)	17 monomers (9 monomers and 4 dimers)
II	51 (51) *^a^*	33 (23)	84 monomers (2 monomers, 16 dimers, 11 tetramers, 1 hexamer)
III	21 (21) *^b^*	31 (17)	52 monomers (52 monomers)
Total	86 (77)	67 (40)	153 monomers

*^a^* For *E. coli* DHOase, among 28 monomers: 4 monomers (all ligand-bound) exhibit loop-in conformations, while 24 monomers (21 ligand-bound) exhibit loop-out conformations. *^b^* Human DHOase represents the only type III enzyme analyzed.

**Table 4:** Distribution of active site loop conformations in ligand-bound DHOase structures across DHOase types.

Ligand	Loop In State	Loop Out State	Total
NCA	10 (Type I: 2, II: 3, III: 5)	6 (Type II: 6)	16
DHO	10 (Type I: 1, III: 9)	13 (Type II: 9, III: 4)	23
DHO/NCA *^a^*	8 (Type III: 8)	2 (Type II: 1, III: 1)	10
5-FOA	4 (Type II: 4)	11 (Type II: 3, III: 8)	15
Orotic acid	0	1 (Type II: 1)	1
Orotic acid/NCA ^*a*^	1 (Type III: 1)	0	1
HDDP	1 (Type II: 1)	1 (Type II: 1)	2
Cacodylate ion	1 (Type I: 1)	0	1
Citrate anion	1 (Type I: 1)	0	1
Acetic acid	2 (Type II: 2)	2 (Type II: 2)	4
Malic acid	30 (Type II: 29, III: 1)	0	30
5-fluorouracil	5 (Type II: 4, III: 1)	0	5
5-aminouracil	4 (Type II: 4)	0	4
Plumbagin	4 (Type II: 4)	0	4
Total	81	36	117

*^a^* The electron density in the structure indicated a mixture of these two compounds, as assigned by the authors.

## Data Availability

The data are contained within the article.

## Supplementary Materials

The supporting information can be downloaded at https://www.mdpi.com/article/10.3390/ijms26199688/s1.
