# Supplementary material for: Conformational Dynamics of the Active Site Loop in Dihydroorotase Highlighting the Limitations of Loop-In Structures for Inhibitor Docking
Source: Int J Mol Sci. 2025 Oct 4;26(19):9688. doi: 10.3390/ijms26199688 (PMC12524618; doi:10.3390/ijms26199688)
Supplement: Supplementary file 1 [file ijms-26-09688-s001.zip › ijms-3874332-supplementary.pdf]

Supplementary data

Supplemental Figure S1. Structural superposition of *Bacillus anthracis* and *Agrobacterium fabrum* DHOases. The structure of *B. anthracis* DHOase (BaDHOase; PDB ID: 3MPG; pale cyan) is superimposed with that of *A. fabrum* (AfDHOase; PDB ID: 2OGJ; green), showing poor structural alignment. Notably, the conserved substrate-binding residue Arg63 (teal) in BaDHOase is replaced by Trp81 (forest green) in AfDHOase.

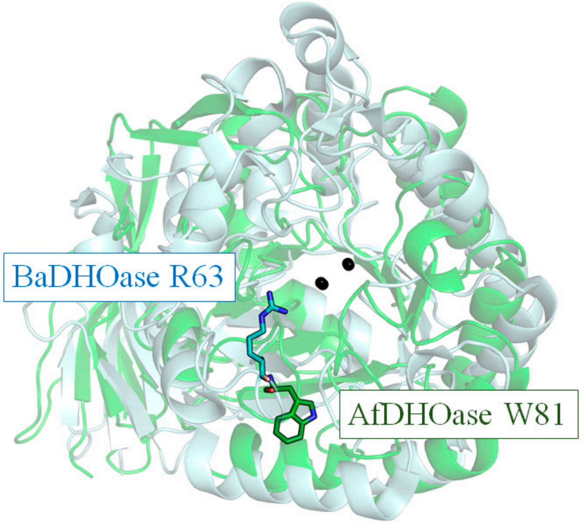

Supplemental Figure S2. Sequence alignment of DHOases from *Aquifex aeolicus*, *Bacillus anthracis*, and *Agrobacterium fabrum*. The amino acid sequences of *A. aeolicus* DHOase (AaDHOase), *B. anthracis* DHOase (BaDHOase), and *A. fabrum* DHOase (WP_010972896.1) were aligned. The conserved substrate-binding arginine residue (R) is highlighted in a red box. In *A. fabrum* DHOase (WP_010972896.1), this position is occupied by tryptophan (W), suggesting potential misannotation of this enzyme as a DHOase—an issue previously identified in *Agrobacterium tumefaciens* C58 as well.

```
WP_010972896.1      MTSGEQAKTPLQAPILLTNVKPVGFGKGASQSSTDILIGGDGKIAAVGSALQAPADTQRI
BaDHOase            ------------MNYLFKNGRYMN-EEGKIVAT-DLLV-QDGKIAKVAENITAD-NAEVI
AaDHOase            -----------MLKLIVKNGYVID-PSQNLEGEFDILV-ENGKIKKIDKNILVP-EAEII
                                   :..*   :.  .    .  *:*:  :***  : . : .  ::: *

WP_010972896.1      DAKGAFISPGWVDLHVHIWHGGTDIS---IRPSECGAERGVTTLVDAGSAGEA-NFHGFR
BaDHOase            DVNGKLIAPGLVDVHVHLREPGGEHKETIETGTLAAAKGGFTTICAMPNTRPVPDCREHM
AaDHOase            DAKGLIVCPGFIDIHVHLRDPGQTYKEDIESGSRCAVAGGFTTIVCMPNTNPPIDNTTVV
                    *.:* ::.** :*:***:.  *   .      : ...  *.**:    .:    :

WP_010972896.1      EYIIEPSRERIKAFLNLGSIGLVACNRVPELRDIKDIDLDRILECYAENSEHIVGLKVRA
BaDHOase            EDLQNRIKE--KAHVNVLPYGAITVRQAG--SEMTDFETLKELGAIAFTDD---GVGVQD
AaDHOase            NYILQKSKS--VGLCRVLPTGTITKGRKG--KEIADFYSLKEAGCVAFTDD---GSPVMD
                    : : :  ..   .  .: . * ::  .     :: *:   .   . * ..:   *  *
```

Supplemental Figure S3. AlphaFold 3.0 structural predictions of DHOases from *Burkholderia cenocepacia* and *Vibrio cholerae*. All five predicted structures of (A) *Burkholderia cenocepacia* and (B) *Vibrio cholerae* provided by AlphaFold 3.0 and shown superimposed here adopt the loop-in conformation. The loop is indicated by a red arrow.

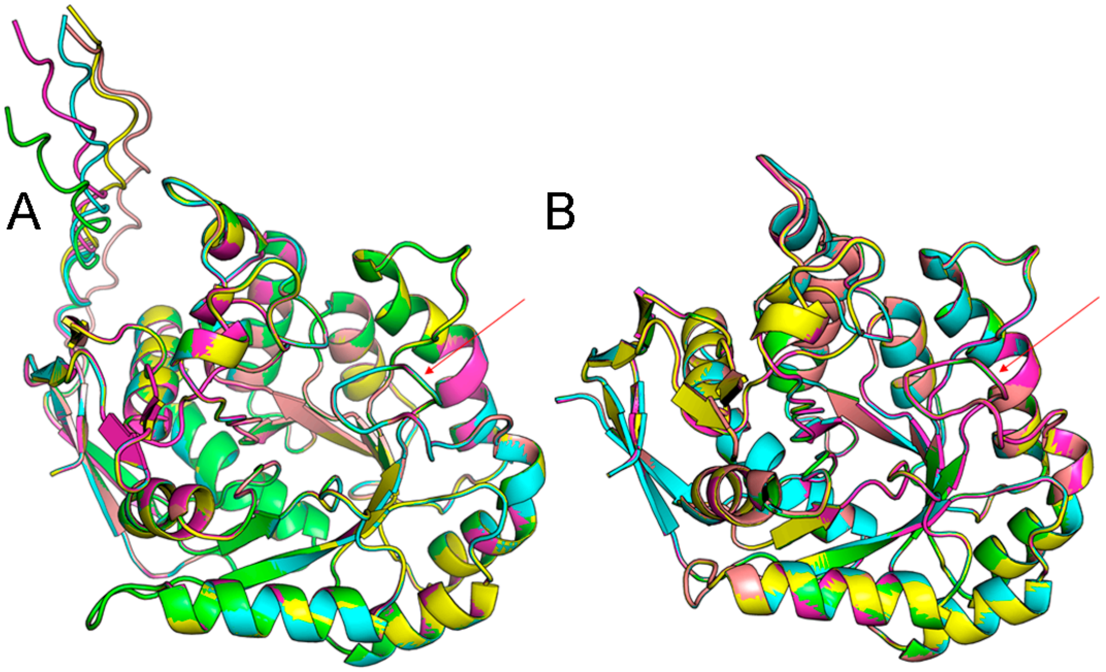

Supplemental Table S1. Docking parameters used for various DHOases.

Receptor	Center_x	Center_y	Center_z	Size_x	Size_y	Size_z
E. coli DHOase (PDB ID 2EG7)						
2eg7_a.pdbqt (loop out)	30.3064	40.0937	80.2873	54.8166450524	50.9262119579	53.7494466782
2eg7_b_f.pdbqt (loop in)	9.1999	14.7938	54.3015	49.5511523437	57.4040186882	55.0819140625
S. enterica DHOase (PDB ID 3JZE)						
3jze_b.pdbqt (loop out)	8.6602	14.5803	9.6279	49.2255176544	54.6808360577	54.7672706413
3jze_a.pdbqt (loop in)	8.6861	14.8605	9.3749	49.0764164352	54.0945221043	55.354446106
C. jejuni DHOase (PDB ID 3PNU)						
3pnu_b.pdbqt (loop out)	44.688	1.9537	-9.5473	59.6565309143	58.2144626236	52.8629675674
3pnu_a.pdbqt (loop in)	23.6719	0.2289	29.3197	60.6225439548	58.5110294914	47.6749306107
Y. pestis DHOase (PDB ID 6CTY)						
6cty_e.pdbqt (loop out)	25.5377	97.3581	150.9526	56.2703956652	47.4757106781	51.0678242493
6cty_a.pdbqt (loop in)	14.8025	56.5752	149.2569	47.2232012463	48.3777827835	61.5916230774
S. cerevisiae DHOase (PDB ID 6L0A)						
6loa_a_del104_108.pdbqt (to mimic the loop-out mode)	-3.1615	25.1058	24.5356	61.5986341095	50.2851005685	60.1623547268

6loa_a.pdbqt (loop in)	-2.9923	25.1585	24.5588	61.5986341095	50.2851005685	60.1623547268
H. sapiens DHOase (PDB ID 4C6C)						
4c6c.pdbqt (loop out)	-16.9054	-19.9869	-11.1334	54.1198386669	51.607291832	56.1546818924
H. sapiens DHOase (PDB ID 8GVZ)						
8gvz.pdbqt (loop in)	-23.9835	19.8851	-11.5313	52.9354623079	52.7841694736	58.8399357224

The grid box for all DHOases used in this study was set to the maximum size to enable docking across the entire protein surface. The exhaustiveness parameter for all docking experiments was set to 8. All available structures of *S. cerevisiae* DHOase exhibit the loop-in conformation. To mimic the loop-out state, residues 104–108 were manually deleted prior to docking (6loa_a_del104_108.pdbqt). This modification enabled successful docking of the compound into the active site of *S. cerevisiae* DHOase.
